# An inside job: how endosomal Na^+^/H^+^ exchangers link to autism and neurological disease

**DOI:** 10.3389/fncel.2014.00172

**Published:** 2014-06-23

**Authors:** Kalyan C. Kondapalli, Hari Prasad, Rajini Rao

**Affiliations:** Department of Physiology, The Johns Hopkins University School of MedicineBaltimore, MD, USA

**Keywords:** SLC9A9, SLC9A6, sodium proton exchanger, autism, Christianson syndrome, ADHD, endosomes, trafficking

## Abstract

Autism imposes a major impediment to childhood development and a huge emotional and financial burden on society. In recent years, there has been rapidly accumulating genetic evidence that links the eNHE, a subset of Na^+^/H^+^ exchangers that localize to intracellular vesicles, to a variety of neurological conditions including autism, attention deficit hyperactivity disorder (ADHD), intellectual disability, and epilepsy. By providing a leak pathway for protons pumped by the V-ATPase, eNHE determine luminal pH and regulate cation (Na^+^, K^+^) content in early and recycling endosomal compartments. Loss-of-function mutations in eNHE cause hyperacidification of endosomal lumen, as a result of imbalance in pump and leak pathways. Two isoforms, NHE6 and NHE9 are highly expressed in brain, including hippocampus and cortex. Here, we summarize evidence for the importance of luminal cation content and pH on processing, delivery and fate of cargo. Drawing upon insights from model organisms and mammalian cells we show how eNHE affect surface expression and function of membrane receptors and neurotransmitter transporters. These studies lead to cellular models of eNHE activity in pre- and post-synaptic neurons and astrocytes, where they could impact synapse development and plasticity. The study of eNHE has provided new insight on the mechanism of autism and other debilitating neurological disorders and opened up new possibilities for therapeutic intervention.

## Genetic complexity of autism and neurodevelopmental disorders

Neurodevelopmental disorders have in common a set of severely affected behavioral features arising from alterations in early brain development that result in life-long disabilities affecting not only the individual but the family and society as a whole (van Loo and Martens, 2007). A characteristic feature of the etiology of neurodevelopmental disorders is a complex interplay between genetics, environment and epigenetic factors. This is exemplified by autism spectrum disorders (ASD) that encompass a heterogeneous collection of neurodevelopmental deficits, with classical autism manifesting in impaired social behavior, repetitive or stereotyped interests, and language delays (Kanner, 1943; Howlin et al., 2000, 2004; Seltzer et al., 2004; Farley et al., 2009). ASD has emerged as a major public health concern worldwide with an unmet need for effective and safe interventions, with the most recent CDC statistics indicating a staggering prevalence of 1 in 68 children (1 in 42 for boys) (Baio, 2014). Individuals with autism are also at high risk for inattention/hyperactivity disorder (14–95%) (Gjevik et al., 2011; Amr et al., 2012; Antshel et al., 2013), intellectual disability (about 40% cases) and seizures (up to 25% cases) (Fombonne, 2003; Canitano, 2007), indicating a common platform in the genetic architecture of these disorders. Within the past two decades, evidence has emerged for strong genetic underpinnings in ASD, estimated at ~70% in monozygotic twins for classic autism and ~90% for the broad spectrum of autistic disorders (Folstein and Rosen-Sheidley, 2001; Landrigan, 2010). However, consistent with the diverse spectrum of clinical presentation is a high degree of genetic heterogeneity, which, together with *de novo* mutations, has hindered analysis of autism susceptibility loci (Liu et al., 2008). In fact, no single mutation accounts for more than ~1% of non-syndromic cases; rather, there appears to be a large number of rare variants (Devlin and Scherer, 2012). Copy number variations and loss of function mutations that affect only one allele highlight the importance of gene dosage impacting the affected pathways (Peters et al., 2013). Although 350–400 genes have been implicated in autism (Iossifov et al., 2012), few have been supported by functional studies at a cellular and molecular level. Future progress in autism research relies not only on identifying new susceptibility genes but also on validating the relevance of candidate genes to the pathogenesis of autism and screening genetic variants for functional changes (Kondapalli et al., 2013). In this review, we will evaluate the emerging evidence implicating a role for endosomal Na^+^/H^+^ exchangers (eNHE), a subgroup of the NHE superfamily, to autism and other neurological disorders. We briefly review relevant studies in model organisms and non-neuronal systems for much-needed functional insight on potential neurological roles of eNHE.

Studies on well-defined neurodevelopmental disorders, such as tuberous sclerosis complex, Fragile X, Timothy, Rett and Angelman syndromes, which have a high co-occurrence of ASD, have provided important clues into etiologic mechanisms of this complex and heterogeneous disorder. One emerging mechanism common to ASD is synaptic plasticity (Delorme et al., 2013) and symptoms of autism emerge in the first few years of life when experience-based modifications of excitatory and inhibitory synapses occur. Neuronal activity triggers changes in transcriptional, translational, signaling and trafficking pathways to regulate learning, memory and adaptive behavior (Flavell and Greenberg, 2008). Dysregulation of any of these crucial steps that control the structure, function and plasticity of the synapse may contribute to the molecular and cellular basis of ASD. A fundamental concept of synaptic physiology is the bidirectional flow of information between astrocytes and neurons. In addition to classic neurotransmitter transmission between pre- and post-synaptic neurons, the astrocytes process, transfer and store information to regulate and respond to synaptic transmission. This three-way communication is described as the tripartite synapse, a phrase originally coined by Philip G. Hayden (Smith, 1994; Araque et al., 1999). Although not localized at the synaptic membrane, eNHE function has been proposed to regulate vesicular trafficking to affect expression and turnover of critical components at the synapse. Therefore, we present a cellular model of eNHE function at each of the three components of the tripartite synapse, including pre- and post-synaptic neurons, and astrocytes. The model incorporates observations drawn from non-neuronal cells and model organisms to propose plausible mechanisms within the context of known pathways in the etiology of neurological disorders.

## eNHE in neurodevelopmental disorders

In recent years, numerous independent studies have implicated NHE6 and NHE9, the two endosomal subtypes (eNHE) of the Na^+^/H^+^ exchanger (NHE) gene superfamily, in multiple neurodevelopmental and neuropsychiatric disorders, including autism, severe X-linked intellectual disability (XLID), epilepsy, addiction and attention deficit hyperactivity disorder (ADHD) (Table 1). The NHE superfamily of cation/proton antiporters control pH and ion composition for a wide range of homeostatic mechanisms in all branches of the tree of life (Brett et al., 2005a). It is well-known that the transport of ions across membranes is crucial for regulation of cellular pH, volume, electrical excitability and energy provision, and thus indispensable for cell survival and proliferation (Casey et al., 2010). A slight alteration in the pH can have profound sequelae: for example, cultured PMC-22 cells enter cell quiescence with a pH_cyt_ perturbation as little as 0.2 units (Musgrove et al., 1987). Furthermore, each membrane-bound organelle must maintain a distinct pH and ionic composition within its lumen that critically impacts its function (Mellman, 1992; Brett et al., 2006). Endocytosis, like many other dynamic cellular processes, requires precise tempo-spatial pH_lumen_ regulation critical for proper processing of receptor ligand complexes, cargo sorting, modulating enzyme activity, membrane protein and receptor recycling, antigen processing, and as a key checkpoint for endosomal sorting machinery and vesicular traffic. Members of the Na^+^/H^+^ exchanger superfamily of membrane transporters provide the range and flexibility to counter fluctuations in cellular and subcellular environment (Brett et al., 2005a).

**Table 1:** **eNHE and neurological disorders**.

**Mutation type**	**Study type**	**Nucleotide change**	**Protein change**	**Phenotype**	**References**
***SLC9A6***					
Missense	Clinical and mutational analysis	c.25G>T (dbSNP: rs201523857)	p.A9S	(1) Angelman-like syndrome, (2) autism spectrum disorder	Fichou et al., 2009;Piton et al., 2011
	Droplet-based multiplex PCR and sequencing	c.563T>C	p.L188P	XLID	Hu et al., 2009
	Exome sequencing	c.1703G>A	p.R568Q	(1) XLID, (2) Schizophrenia	Tarpey et al., 2009(^*^);Piton et al., 2011(^*^);Santoni et al., 2014(^*^)
Non-sense	Brain MRI, clinical and mutational analysis	c.916C>T	p.Q306X	XLID, Christianson type and retinitis pigmentosa	Mignot et al., 2013
	Brain MR spectroscopy, clinical and mutational analysis	c.1219C>T	p.Q407X	XLID, Christianson type	Schroer et al., 2010
	Linkage analysis, DNA sequencing and biochemical investigations	c.1498C>T (dbSNP: rs122461162)	p.R500X	(1) XLID, (2) Angelman-like syndrome	Gilfillan et al., 2008(^*^);Tarpey et al., 2009(^*^); Schroer et al., 2010
	Exome sequencing	c.1639G>T	p.Glu547X (truncation of tail)	XLID	Schuurs-Hoeijmakers et al., 2013
Splicing	Brain MRI, Clinical and mutational analysis	c.680+1G>T		XLID, Christianson type	Riess et al., 2013(^*^)
	Exome sequencing	c.1042-1C>T (dbSNP: rs149044510)		XLID	Piton et al., 2013
	Brain MRI, clinical and mutational analysis	c.526+1G>A		XLID, Christianson type	Bosemani et al., 2013
	Brain MRI, EEG, clinical and mutational analysis	c.1151-1G>A		XLID, Christianson type and electrical status epilepticus during slow-wave sleep (ESES)	Zanni et al., 2014
Small deletions	Linkage analysis, DNA sequencing and biochemical investigations	c.860 865delAAAG TG	p.G287 S288del	(1) XLID, (2) Angelman-like syndrome	Gilfillan et al., 2008(^*^)
	Linkage analysis, mutation screening, electron microscopy examination, biochemical investigations	c.1109_1117delGG AGTACCT	p.W370 T372del	XLID, autism spectrum disorder, tau deposition and stereotyped, repetitive hand movements mimicking Rett syndrome	Garbern et al., 2010
	Clinical, mutational and biochemical analysis	c.441delG	p.S147MfsX9	Angelman-like syndrome	Takahashi et al., 2011
	Linkage analysis, DNA sequencing and biochemical investigations	c.608_609delAT	p.H203LfsX60	(1) XLID, (2) Angelman-like syndrome	Christianson et al., 1999;Gilfillan et al., 2008(^*^);Tarpey et al., 2009(^*^)
	Linkage analysis, DNA sequencing and biochemical investigations	c.603+1delGTAA	p.V176 R201del	XLID	Gilfillan et al., 2008(^*^);Tarpey et al., 2009(^*^)
Small insertions	Brain MRI, Clinical and mutational analysis	c.1560dupT	p.T521YfsX23	XLID, Christianson type	Riess et al., 2013(^*^)
Gross deletions	Array comparative genomic hybridization analysis	314 kb involving NHE6 exons 15-16, FHL1, MAP7D3 and GPR112 genes		XLID, Christianson type	Tzschach et al., 2011
	Array comparative genomic hybridization analysis	9 kb partial		XLID	Whibley et al., 2010
Duplications	MLPA analysis, fluorescence *in situ* hybridization, X-chromosome-specific aCGH, breakpoint mapping by quantitative PCR	2.8 Mb involving 24 known genes including NHE6		Intellectual disability, short stature, microcephaly, and hypopituitarism	Madrigal et al., 2010
Non-coding SNPs	Clinical and mutational analysis	c.1538+8G>A (dbSNP: rs6654310)		Psychomotor delay and stereotypes	Fichou et al., 2009
		c.1725-4G>A		Autism	
***SLC9A9***					
Missense	Homozygosity mapping and mutational analysis	c.349C>A (dbSNP: rs186296463)	P117T	Autism with epilepsy	Morrow et al., 2008
		c.526G>A	V176I	Autism without epilepsy	
		c.707T>C (dbSNP: rs113649536)	L236S	Autism without epilepsy	
		c.1312T>C	S438P	Autism with epilepsy	
		c.1486G>A (dbSNP: rs111291437)	D496N	Autism with epilepsy	
		c.1825C>A	Q609K	Autism without epilepsy	
	Candidate gene association analysis	c.1765A>G (dbSNP: rs2289491)	I589V	Attention deficit hyperactivity disorder	Brookes et al., 2006
Non-sense	Homozygosity mapping and mutational analysis	c.1267C>T (dbSNP: rs121912597)	R423X	Autism with epilepsy	Morrow et al., 2008
Splicing	Homozygosity mapping and mutational analysis	c.1524+3A>G		Autism without epilepsy	Morrow et al., 2008
Deletions	SNP arrays and mutational analysis	12 kb partial		Autism spectrum disorder	Ben-David et al., 2011
	Homozygosity mapping and mutational analysis	Non-coding 5′ NHE9 and entire DIA1 gene		Autism	Morrow et al., 2008
Complex rearrangements	Biochemical investigations, association and linkage studies	Inv (3)(p14:q21)		Attention deficit hyperactivity disorder	de Silva et al., 2003
Non-coding SNPs	Candidate gene association analysis	Multiple SNPs		Attention deficit hyperactivity disorder	Brookes et al., 2006
	GWAS	Multiple SNPs		Attention deficit hyperactivity disorder	Lasky-Su et al., 2008
	Family based GWAS	dbSNP: rs9810857		Attention deficit hyperactivity disorder	Mick et al., 2010
	GWAS, clinical and MRI analysis	c.534-13829C>T (dbSNP: rs9828519)		Multiple sclerosis, non-response to interferon-ß	Esposito et al., 2013
	Network based GWAS	c.1604+13022A>G (dbSNP: rs6775025)		SNP with lowest *p*-value for smoking initiation	Vink et al., 2009
		c.378+8918C>G (dbSNP: rs4839669)		SNP with lowest *p*-value for current smoking	
	ADHD clinical assessment tools, genotyping	c.^*^444C>A (dbSNP: rs1046706)		ADHD-related deficits on impulsiveness and hyperactivity	Markunas et al., 2010
		c.649+6371C>T (dbSNP: rs2360867)		ADHD-related deficits on commission errors	

*Survey of studies implicating eNHE in a range of neurological disorders*.

Abbreviations: del, deletion; fs, frame-shift; Inv, inversion; SNP, single nucleotide polymorphism; dbSNP, NCBI SNP database entry; XLID, X-linked intellectual disability; X, termination; (

* *) Mutations are referred to in relation to longer NHE6.1 isoform (NP_001036002.1)*.

In humans, NHE genes are part of the Solute Carrier 9 (*SLC9*) cluster that includes 13 different isoforms organized into distinct divisions on the basis of phylogenetic relations. However, despite abundant and wide spread expression in the brain, only a specific subset of the *SLC9* gene family has been linked to autism and neurodevelopmental disorders. To understand how eNHE may contribute to these disorders, a brief overview of the evolutionary origins and cellular role of NHE isoforms is useful. Readers are directed to Donowitz et al. (2013) for a more detailed, up to date review of cation/proton exchangers.

**Figure 1:**
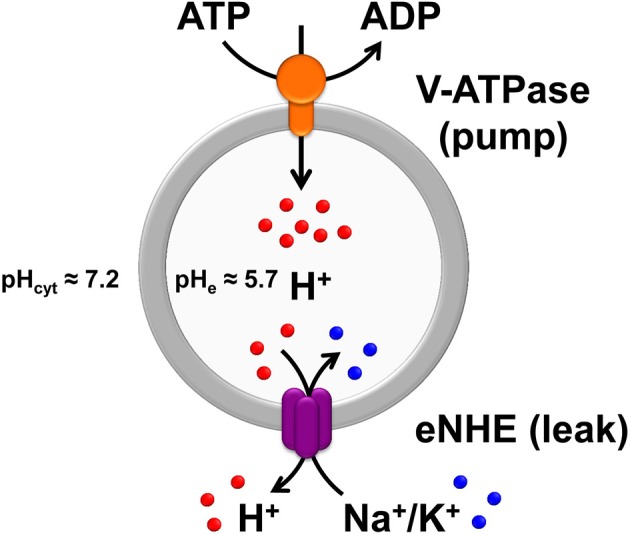
**The Pump-Leak hypothesis of endosomal pH regulation**. Endosomal pH is precisely tuned by a combination of proton pumping or acidification through the V-ATPase and proton leaking or alkalization via endosomal Na^+^(K^+^)/H^+^ exchangers (eNHE), both evolutionarily conserved from yeast to plants and mammals. In mammalian cells, under physiological conditions, the endosomal pH is acidic (≈5.7) relative to the cytoplasmic pH (≈7.2). As per the pump-leak hypothesis, the H^+^ gradient generated by the V-ATPase (V-type H^+^-ATPase) drives secondary active transport by eNHE, resulting in cation (Na^+^ or K^+^) sequestration coupled to removal of protons from the compartmental lumen. From this model it is intuitive that eNHE activity would provide the range and flexibility to counter fluctuations in subcellular environment. Also, loss-of-function mutations in eNHE would cause hyperacidification of endosomal lumen, as a result of imbalance in pump and leak pathways, impacting cargo processing, turnover, and trafficking. In addition to the intricate balance between proton pump and leak pathways, counter-ion (anion and cation) conductances (not shown) also contribute to pH homeostasis. The pH values indicated in the figure were collected from published literature (Casey et al., 2010; Kondapalli et al., 2013).

Members of the electroneutral NHE clade (*SLC9A*) comprise 9 isoforms that reside on the plasma membrane (NHE1–5; *SLC9A1–5*) or in distinct intracellular compartments (NHE6–9; *SLC9A6–9*) including Golgi (NHE8; *SLC9A8*) and *trans*-Golgi network (NHE7; *SLC9A7*), and in various endosomes (NHE6; *SLC9A6*, and NHE9; *SLC9A9*) (Ohgaki et al., 2011; Donowitz et al., 2013). Furthermore, the plasma membrane NHE (pNHE) may be stably resident at the cell membrane (NHE1, 2, and 4) or recycle there and back (NHE3 and 5). Each isoform is thought to play a distinct cellular role. In general, plasma membrane NHE isoforms regulate cytoplasmic pH whereas the effect of the intracellular isoforms is primarily on the compartmental lumen. Plasma membrane NHE function by coupling to the Na^+^ electrochemical gradient established by the ubiquitous sodium pump, moving Na^+^ ions into the cytoplasm in exchange for removal of H^+^. Thus, apically localized Na^+^/H^+^ exchangers, like NHE3, are important in sodium reabsorption in the kidney and gut (Donowitz et al., 2013). In contrast, active transport by endosomal NHE is driven by the H^+^ gradient generated by the V-type H^+^-ATPase, resulting in cation (Na^+^ or K^+^) sequestration coupled to removal of protons from the compartmental lumen (Figure 1). Of note, the plasma membrane and endosomal subtypes have different ion selectivity, with the former being Na^+^ selective, whereas most intracellular isoforms transport both K^+^ and Na^+^ ions (Ohgaki et al., 2011). In this way, eNHE activity modulates pH within endosomes and various secretory organelles, which has been linked to cargo processing, turnover and trafficking, as described ahead.

## Functional insights on eNHE from non-neuronal systems

A strong body of literature relating to eNHE activity in non-neuronal model systems highlights their key role in many fundamental cellular functions including endosomal pH regulation and controlling vesicular traffic. These valuable insights can applied toward deciphering the role of eNHE in neuronal cells where they play a role in the etiology of neurological disease. Intracellular Na^+^/H^+^ exchangers have been identified and studied in model systems ranging from *Saccharomyces cerevisiae* and other yeasts, to *Arabidopsis* and other plants, the nematode *Caenorhabditis elegans* and flies, including *Drosophila* and mosquito, and more recently using knockout models in mouse. Each of these systems has provided unique insight that may be extrapolated to human counterparts. From yeast, it is apparent that endosomal NHE largely impacts intracellular trafficking pathways by modulating luminal pH (Brett et al., 2005b). In plants, vacuolar NHE modulate flower color by altering luminal pH (Fukada-Tanaka et al., 2000), confer salt tolerance and resistance to osmotic stress, regulate stomatal movements, and affect leaf development (Apse et al., 1999; Andres et al., 2014), while eNHE orthologs that localize to endosomal compartments in the plant cell have been postulated to play a role in trafficking and regulation of vesicular function (Pardo et al., 2006).

A useful strategy to obtain preliminary information on ion selectivity and pH dependence of mammalian NHE is by complementing knockout phenotypes of the orthologous gene (*NHX1*) in yeast. We found that NHE6 and NHE9 both conferred salt tolerant growth in medium adjusted to pH 7–7.5, but not at acidic pH (Hill et al., 2006). Yeast NHX1, however, was able to confer salt tolerance over a wide range of pH. Since alkaline medium was found to elevate vacuolar pH but not cytoplasmic pH (Brett et al., 2011), these observations hint at a potential role of luminal pH in regulating mammalian endosomal NHE activity. The ion-selectivity of NHE6 and NHE9 was evaluated using this heterologous system. Both genes complemented host sensitivity to Na^+^ and K^+^, but not to Li^+^ or Rb^+^. Yeast NHX1 differs in the ability to also transport Rb^+^, as shown by relative tolerance to RbCl toxicity and ^86^Rb transport (Brett et al., 2005b).

Relative to work done on the plasma membrane isoforms, functional characterization of mammalian endosomal NHE has been conspicuously slow. This is in part due to the methodological limitations. Standard assays of plasma membrane-NHE activity which follow pH-dependent recovery of BCECF fluorescence after acidification of cytosol, consistently failed to detect endosomal-NHE function, whereas measurement of luminal pH using compartment specific pH-sensitive GFP constructs often show modest changes, possibly in part due to the buffering role of GFP itself inside small vesicles. In COS7 cells, Nakamura et al. observed an elevation of 0.4 pH units in recycling endosomes upon overexpression of NHE9 (Nakamura et al., 2005). In the latter study, no effect of NHE6 overexpression was observed possibly due to non-overlapping distribution with the pH probes. Depending on cell type, there is a partial overlap in the distribution of NHE6 and 9 in endosomes, such that luminal acidification has been observed upon NHE6 knockdown either singly (HepG2 cells and HeLa cells) (Ohgaki et al., 2010; Xinhan et al., 2011) or in combination with NHE9 knockdown (HeLa cells) (Roxrud et al., 2009). In an elegant study of auditory hair cells, Hill et al. (2006) demonstrated a role for NHE9 in pH homeostasis in the stereocilia. They showed that Ca^2+^/H^+^ exchange by plasma membrane Ca^2+^-ATPase resulted in rapid acidification of the hair bundles and that electroneutral K^+^/H^+^ activity by NHE9 exploited the K^+^ rich endolymph surrounding hair bundles to prevent overacidification. In these specialized cells, NHE9 was localized to the plasma membrane of stereocilia, in addition to vesicles in the cell body and contributed to pH homeostasis of the hair bundles, independently of the cell body (Hill et al., 2006).

The yeast model has taught us that effects of NHX1 on trafficking are robust and easy to monitor. Indeed, this has been borne out by more recent and insightful studies investigating the role of mammalian eNHE in trafficking: Muro et al. showed that in endothelial cells cargo becomes associated with NHE6-positive compartments 3 h following endocytosis. Furthermore, they showed that monensin, an ionophore known to abolish Na^+^/H^+^ gradients, blocks lysosomal delivery and reroutes traffic to the surface (Muro et al., 2008). Using transferrin uptake assays, NHE6 activity was implicated in clathrin-mediated endocytosis (Xinhan et al., 2011). In the polar hepatocyte cell model, HepG2, Ohgaki et al. showed colocalization of NHE6 to recycling endosomes (both sorting endosomes and the subapical compartment) where it was required for retention of apical membrane components and maintenance of cell polarity. Following isoform-specific knockdown and overexpression of NHE6, they observed a decrease and increase of endosomal pH, respectively, that ultimately affected the polarized distribution of membrane lipids at the apical surface. In addition, they clarified that maintenance, but not biogenesis, of the apical membranes is regulated by NHE6 (Ohgaki et al., 2010).

## Insights from interacting partners

**Figure 2:**
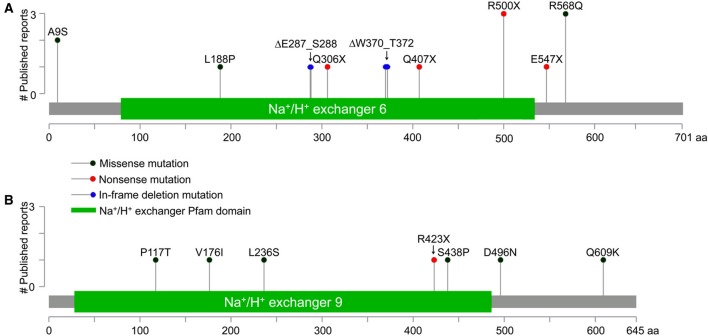
**Gene Distribution of patient mutations in NHE6 and NHE9. (A,B)** The distribution of NHE6 **(A)** and NHE9 **(B)** mutations (missense, non-sense, and in-frame deletions) in N-terminal transmembrane domain and the C-terminal regulatory cytoplasmic domain, listed in Table 1, are displayed. The x-axis indicates amino acid locations of NHE6 and NHE9 proteins, while the y-axis displays the number of published literature reports. All mutations in NHE6 are referred to in relation to longer NHE6.1 isoform (NP_001036002.1).

The presence of several human disease mutations in the C-terminal tail of eNHE point to an important functional role of this domain (Figure 2; Table 1). For instance, Arg568Gln in the C-terminus of NHE6.1 isoform (Arg536Gln in the shorter NHE6.0 isoform) was identified in three independent reports: two in males with intellectual disability (hemizygous) and one in a female with schizophrenia (heterozygous) (Tarpey et al., 2009; Piton et al., 2011; Santoni et al., 2014). The cytoplasmic C-terminal tail of the NHE lies outside the membrane-embedded transport domain and is not considered essential for ion transport. However, the C-terminal domain may act as a scaffold for binding of multiple proteins that regulate transport kinetics and trafficking. Therefore, the role of interacting proteins at the C-terminus of eNHE is of special interest to understanding the molecular and cellular basis of autism and related neurological disorders.

Hydropathy analysis of the Na^+^/H^+^ exchangers predicts a common membrane topology shared by all isoforms (NHE1–9): an N-terminal hydrophobic membrane-embedded domain consisting of 12 transmembrane segments and a hydrophilic C-terminal domain, facing the cytoplasm. Much of the conservation between NHE isoforms (up to 70% in amino acid similarity) is restricted to the N-terminal transporter domain, with the C-terminus being more divergent. For example, in the C-terminus, the amino acid identity between NHE1 (resident, plasma membrane isoform) and the eNHE is ~8–10% and sequence similarity is ~37–41%, whereas within the two endosomal isoforms the identity and similarity are ~42 and 87%, respectively. This suggests that distinct interactions at the C-terminus probably have an effect on the localization and regulation of the eNHE. There are many published studies on the role of interacting partners of plasma membrane NHE, particularly NHE1 and NHE3, linking transporter function to cytoskeleton and signal transduction (Donowitz et al., 2013). In principle, some of the known interacting partners of well-studied pNHE, for instance an autism-associated scaffolding protein Shank2 shown to interact and regulate NHE3 activity (Han et al., 2006), might be useful candidates in the search for binding partners of eNHE.

Specific interacting partners of the eNHE have been reported. RACK1 (Receptor for Activated C-Kinase 1), previously shown to be an adaptor and a scaffold protein, interacts with NHE 6, 7, and 9 *in vitro* and was shown regulate the cellular localization of NHE6 (Ohgaki et al., 2008). While the bulk of NHE6 is localized in early/recycling endosomes, it appears transiently on the plasma membrane (Brett et al., 2002). Surface biotinylation studies showed that knockdown of RACK1 decreased surface expression of NHE6 without changing total protein levels. This decrease on the surface was accompanied by elevation of endosomal luminal pH and decreased uptake of transferrin, a marker for the recycling pathway. The only other published interaction of NHE6 is with Angiotensin II receptor subtype 2 (AT2). Angiotensin II (Ang II) has been shown to regulate sodium homeostasis by binding to its receptors AT1 and AT2, which mediate opposing signaling mechanisms. Using AT2 as bait in a yeast two-hybrid approach, Pulakat and coworkers identified a peptide of NHE6. They showed that AT2 co-immunoprecipitated with hemagglutinin tagged-NHE6 when expressed in a human breast cancer cell line, MCF-7, but only under conditions where the cells were treated with Ang II, suggesting that the interaction is ligand mediated (Pulakat et al., 2005). A number of human mutations have been reported in the AT2 including some involving NHE6 binding region of AT2. Intriguingly, human mutations in AT2 are also reported to result in XLID and epilepsy and might have overlapping clinical features with NHE6 mutations (Vervoort et al., 2002; Takeshita et al., 2012). In the case of NHE9, co-immunoprecipitation of tagged constructs expressed in HEK293 cells revealed an interaction with the calcineurin homologous protein CHP, previously shown to bind to the C-tail of pNHE isoforms (Lin and Barber, 1996; Zhang-James et al., 2012). Interestingly, a C-terminal NHE9 variant V512G/K534R found in a rat model of inattentive behavior showed a 2-fold increase in binding CHP, although the functional implication of this remains to be determined (Zhang-James et al., 2012).

## Patient mutations in eNHE: gene distributions and structural insights

A plethora of genetic studies has uncovered numerous clinically relevant patient mutations in *SLC9A6* and *SLC9A9* genes, encoding NHE6 and NHE9 respectively. These span the gamut of missense and non-sense mutations, both large and small deletions and insertions, splice site mutations, microduplications and non-coding single nucleotide polymorphisms, and are listed in Table 1. Gene alterations are distributed throughout the open reading frame, localizing within the N-terminal membrane spanning transporter domain, and the cytoplasmic C-terminal tail (Figure 2). Although invaluable in training a spotlight on the eNHE, genetic analyses and gene sequencing approaches alone do not provide definitive functional insight in the absence of structure-function information. Functional evaluation of disease-associated eNHE variants will be essential to predict clinical outcome, as a prerequisite to personalized therapy in patients with autism and other neurological diseases. Thus, a given alteration may be a harmless polymorphism or causal to the disease. Mutations may exert their effect through loss or a gain of function and in the case of single allele alterations these may exert their impact through haploinsufficiency or a dominant effect. Therefore, it is imperative to validate patient mutations using a combination of structure-based and functional tools. In the next section we will briefly discuss model-structures of eNHE that could be used as a template for structure-driven assessment to predict functionally significant gene variants.

**Figure 3:**
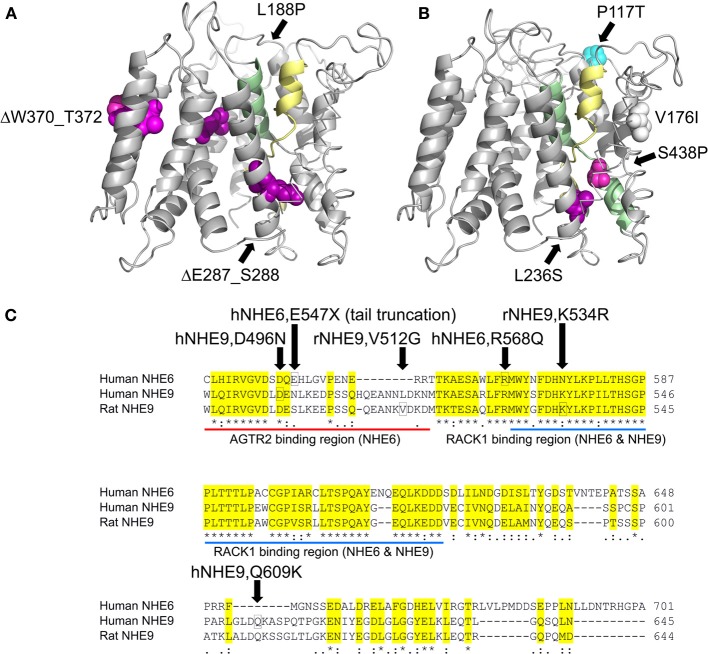
**Model-Structures of NHE6 and NHE9 with patient mutations**. **(A,B)** Model of NHE6 **(A)** and NHE9 **(B)** are based on methods described in detail elsewhere (Landau et al., 2007; Kondapalli et al., 2013). Side views of the membrane embedded N-terminal transporter domains are shown, with the cytoplasm below. The TM4–TM11 assembly (colored yellow and green, respectively) is key to the antiport mechanism, and consists of extended segments of the two helices, crossing each other in the middle of the membrane. For clarity, TM3 backbone is shown in stick configuration and a part of the TM1–2 loop of NHE9 was omitted. Positions in which clinical mutations were detected are marked, shown as all-atom spheres and colored according to their ConSurf evolutionary conservation scores (Ashkenazy et al., 2010, http://consurf.tau.ac.il/) as follows: strongly conserved residues (magenta), weakly conserved (white), non-conserved (blue). Many of the conserved sites that show substitutions are situated in highly packed regions, including the vicinity of the TM4–TM11 assembly region. P117 of NHE9, on the other hand, is located in the very long and variable loop connecting TM1 and TM2. A minor substitution to Ile was found in V176 of NHE9, a moderately-conserved hydrophobic residue that is oriented toward the lipid bilayer. Three autism-associated variants in NHE9 (L236S, S438P, V176I; Table 1) have been experimentally determined to be loss of function mutations (Kondapalli et al., 2013), the remainder remain to be screened for changes in function. **(C)** Sequence alignment of C-terminal tail extensions of human NHE6, human NHE9, and rat NHE9. The positions of human NHE6 and NHE9 disease variants in the C-terminal tail are boxed (Table 1). Missense variants reported in rat models of attention deficit/hyperactivity disorder are also indicated (Zhang-James et al., 2011). Highly conserved RACK1 binding region in the C-terminus of NHE6 and NHE9 and the Angiotensin II receptor (AT2) interacting region in the juxtamembrane portion of the NHE6 C-terminus are displayed.

Three dimensional protein structures are a requisite first step toward interpreting functional effects of gene variants in eNHE. Although atomic-level structures of mammalian Na^+^/H^+^ exchangers have not been experimentally derived, crystal structures of bacterial CPA orthologs serve as templates for predictive model-structures based on a common protein fold, supported by evolutionary conservation analysis and empirical data. The low sequence identity (~15%) across large evolutionary distances separating bacterial and mammalian orthologs makes the modeling challenging, requiring state of the art approaches to construct sequence alignments, which are then used to build three dimensional homology models of NHE isoforms based on the template structure of *E. coli* NhaA (Figures 3A,B) (Hunte et al., 2005; Kondapalli et al., 2013). More recently, the crystal structure of the *T. thermophiles* NapA antiporter was obtained in a distinct outward-facing conformation (Lee et al., 2013), providing further evidence for the broadly accepted alternating access model of antiport. Together, the structures reveal a unique two-domain rocking bundle of twelve transmembrane helices with a dimerization domain showing extensive helix contacts between adjacent monomers. The translocation domain contains a pair of distinctive crossing helices that are partially unwound in the middle of the bilayer, the TM4–TM11 assembly (Figures 3A,B) proposed to provide a delicate electrostatic balance for nearby charged residue(s) that form the putative cation binding site. Importantly, the most highly conserved residues in the model-structures localize to helix interfaces within the transmembrane core, whereas the more variable residues lie in loops or face the lipid bilayer (Landau et al., 2007). Substitutions of highly conserved residues, particularly within structures important for transport function, are likely to disrupt transport function.

Missense mutations and in-frame deletions in *SLC9A6* and *SLC9A9* from the studies listed in Table 1 have been mapped onto the model-structures (Figures 3A,B). In the absence of an equivalent domain, the C-terminal tails of mammalian NHE could not be modeled, and are represented in Figure 3C. Other variants that introduce non-sense (stop) codons within the membrane embedded transporter domain are not indicated, and are expected to result in clearance of truncated polypeptides or in non-sense-mediated transcript decay. While some patient-associated variants introduce non-conservative substitutions within highly conserved domains, others lie in more variable regions of the transporter.

With the advent of personal genomics on the horizon, and the prevalence of many rare variants in individual genotypes, it will be important to have in place defined strategies to evaluate functional consequences of amino acid changes in eNHE that may be linked to neurodevelopmental disorders. One such strategy involves phenotype complementation in a yeast expression system, using the yeast ortholog as a surrogate in an initial screen for function. Functional evaluation of a subset of missense mutations reported for NHE9 revealed that changes in conserved residues could readily be modeled in yeast and gave consistent loss-of-function effects in both yeast and murine astrocyte models (Kondapalli et al., 2013). However, a substitution in a more variable region that resulted in functional deficiency in the astrocyte cell model, failed to show difference from wild type protein in yeast. Although more extensive testing of patient variants is warranted, this limited study suggested that residues with an evolutionary score of 5 or better (on a scale of 1–8, with 8 being most conserved; Kondapalli et al., 2013) could be readily amenable to rapid and inexpensive functional evaluation using phenotype screening in yeast. Less conserved substitutions may have to be evaluated in a neurobiological model. Thus, structure-driven assessment of gene variants in a simple yeast model complemented with neuronal/astrocytic models would provide a rapid, inexpensive and accurate functional screen for eNHE gene variants to distinguish between harmless polymorphisms and disease-associated mutations.

## Patient mutations in eNHE: clinical features

Patient mutations in eNHE present an unexpectedly large range of neurodevelopmental disorders, with distinct but overlapping phenotypes. The complexity of clinical presentation has remained largely unexplained at a functional level, particularly in the context of neurobiology and function of the synapse. Herein, we seek to consolidate all relevant clinical data on eNHE that span a range of patient symptoms as a starting point for genotype-phenotype correlations and to aid clinical decision making.

### NHE6 (SLC9A6)

*SLC9A6*, the gene encoding NHE6, is one of six most recurrently mutated loci in patients with XLID and has been linked to autism comorbid with seizures (Tarpey et al., 2009). Mutations in the *NHE6* gene commonly present with prominent neurological phenotypes associated with syndromic autism that can be broadly characterized into four overlapping clinical categories, summarized in Table 1 and described below.

- **Christianson syndrome:** Christianson et al. first described a XLID syndrome localized to Xq24-q27 in a South African family presenting with autistic features and cerebellar atrophy (OMIM 300243)^1^ (Christianson et al., 1999). A follow-up study on this family located the causative NHE6 mutation and reported three additional families with the disorder (Gilfillan et al., 2008). Christianson syndrome is characterized by severe developmental delay, early onset seizures, ataxia, hypotonia, and microcephaly in males. At least fifteen unique mutations in *SLC9A6* have been reported thus far (Schroer et al., 2010; Stromme et al., 2011; Tzschach et al., 2011; Mignot et al., 2013; Riess et al., 2013) (Table 1). A 314 kb deletion in the Xq26.3 region affecting NHE6 and FHL1 genes in a 2-year-old boy was associated with symptoms of severe intellectual disability, absent speech, ataxia, and epilepsy likely from functional deficits in NHE6, whereas FHL1 gene disruption was associated with later onset of muscular dystrophy (Tzschach et al., 2011). A mutation in *SLC9A6* in a 22-year-old male introduced a stop codon at amino acid residue Gln306 (Mignot et al., 2013). Pervasive developmental disorder ^2^ followed by profound intellectual disability was observed in this patient, as well as retinitis pigmentosum, previously unreported in Christianson syndrome. A study showing novel *SLC9A6* gene mutations in Christianson syndrome males noted signs of Parkinsonism in obligate carrier females from the same families, suggesting a predisposition to late-onset neurodegenerative disorders (Riess et al., 2013).
- **Angelman-like syndrome:** Several clinical syndromes share overlapping clinical and behavioral phenotypes with Angelman Syndrome (AS), and thus are sometimes misdiagnosed (see Seltzer and Paciorkowski, 2014; Tan et al., 2014 for detailed review). Recognition of the overlap in the clinical spectra of Angelman-like syndromes is important for differential diagnosis and clinical decision making. Patients with NHE6 mutations share several clinical features with Angelman syndrome including developmental delay, postnatal microcephaly, language impairment, happy demeanor, frequent laughter, drooling, ataxia, seizures, and stereotypic behaviors (Fichou et al., 2009; Schroer et al., 2010). Reports in literature have described mutations in NHE6 in patients clinically diagnosed with AS. While most cases of AS are linked to the 15q11-q13 region of the chromosome associated with disruption of *UBE3A* gene expression, 10–15% of the cases are due to alternative genetic mechanisms (Williams et al., 2001). Patients with AS share phenotypes with Rett syndrome, characterized by mutations in *MECP2* gene. Gilfillan et al. investigated candidate genes in patients with phenotypes that resembled Angelman syndrome but did not have mutations in the *UBE3A* or *MECP2* genes (Gilfillan et al., 2008). Linkage analysis and DNA sequencing studies identified mutations disrupting *SLC9A6* in patients from multiple families (Table 1). These patients exhibit a slow progression of symptoms, with resemblance to Angelman syndrome being more striking at a younger age. Proton magnetic resonance spectroscopy data from an effected patient showed elevated levels of glutamate-glutamine complex in the basal ganglia which could be associated with a variety of the clinical neurological abnormalities, including epilepsy and cerebellar degeneration (Gilfillan et al., 2008). Unlike classic Angelman, which is characterized by obesity, patients with mutations in *SLC9A6* are lean. They also show a distinct EEG pattern (rapid background frequency of 10–14 Hz vs. slow frequency of 1.5–3 Hz) (Gilfillan et al., 2008). It has recently been proposed that cerebellar atrophy and/or hyperintensity of cerebellar cortex could serve as specific neuroimaging hallmarks of NHE6 mutations, distinct from AS patients (Bosemani et al., 2013). The genetic and molecular basis of the overlap in phenotypes between NHE6 mutations and AS remains largely unknown. Intriguingly, in NHE6 knockout mice, there is extensive degeneration in the hippocampal neurons and in cerebellar Purkinje cells, sites of expression of the maternal allele of *UBE3A* (Albrecht et al., 1997; Stromme et al., 2011). It is worth noting that both NHE6 and UBE3A are activity-regulated MEF2 target genes that may regulate activity-dependent synaptic plasticity during neural network development (Flavell et al., 2008).
- **Corticobasal degeneration with tau deposition:** Garbern et al. described a family with intellectual disability accompanied by virtual absence of speech, autism spectrum disorder, epilepsy, late-onset ataxia, weakness, and dystonia (Garbern et al., 2010). Intriguingly, affected males in the family did not show Angelman-like phenotype. Instead, several of the affected males had stereotyped, repetitive hand movements, a phenotype mimicking Rett syndrome (Chahrour et al., 2008). The disorder mapped to an in-frame 9 base pair deletion in *SLC9A6*. Histopathological examination of post-mortem brain tissue from these patients showed wide spread neuronal loss, gliosis and deposits of the hyperphosphorylated microtubule binding protein tau (predominantly 4R type) in tangles and inclusions in neurons and glia. Loss of NHE6 function is the likely molecular mechanism for the cellular dysfunction associated with this mutant (Ilie et al., 2013).
- **Epilepsy:** Epilepsy is a common comorbidity seen in autism and intellectual disability (Canitano, 2007). Grand mal epilepsy has been reported in 87.5% of patients from a family with NHE6 mutation (Christianson et al., 1999). Independent observations of increased levels of glutamate-glutamine complex in the basal ganglia in the basal ganglia in MR spectroscopy analysis of patients with NHE6 mutations suggest that increased glutamate concentration in the brain might induce seizures (Gilfillan et al., 2008; Schroer et al., 2010). NHE6 mutations are associated with electrical status epilepticus during slow-wave sleep (ESES), a form of severe epileptic disorder (Zanni et al., 2014). Controlling seizures in patients with intellectual disability is known to improve clinical outcomes and quality of life (Jozwiak et al., 2011). Given that neuronal pH dysregulation has been linked to origin and progression of seizure activity (Somjen, 1984), correction of endosomal pH with drugs that alkalinize endosomal compartments might be a useful therapeutic strategy to limit the progression of the disease.

In addition, to gene deletions and mutations, microduplications in Xq26.2-q26.3 involving NHE6 have been reported in patients with intellectual disability, short stature, microcephaly, and hypopituitarism (Madrigal et al., 2010). Similar clinical presentations have been described with MECP2 mutations, wherein both loss of function mutations and increased gene dosage resulting from gene duplications are associated with prominent neurological phenotypes including intellectual disability (Chahrour et al., 2008; Peters et al., 2013).

### NHE9 (SLC9A9)

A range of clinical presentations have been linked to mutations in the *SLC9A9* gene expressing NHE9 (Table 1; Figure 2). Mutations in NHE9 present with distinct albeit overlapping phenotypes to those of NHE6, suggesting unique, non-compensating functions of the two isoforms.

- **Autism:** The susceptibility to autism-16 (AUTS16) locus on chromosome 3q is associated with mutations in the NHE9 gene, *SLC9A9* (OMIM: 613410)^3^. Homozygosity mapping, microarray, and sequencing data from families of autism patients with shared ancestry showed a chromosomal deletion in a potentially regulatory non-coding region upstream of *SLC9A9* (Morrow et al., 2008). Other autism associated deletion mutations involving NHE9 have been also reported in the literature (Ben-David et al., 2011; Wagle and Holder, 2014). Furthermore, in non-consanguineous autistic pedigrees several rare coding variants and a non-sense mutation truncating the last predicted extracellular loop of the 10–12 membrane-spanning NHE9 protein were identified in patients with autism and epilepsy (Morrow et al., 2008). We evaluated three representative autism-associated variants for NHE9 function using model structure-based evolutionary conservation analysis, phenotype screening in yeast and pH-dependent analysis of trafficking in primary mouse astrocytes (Kondapalli et al., 2013). The study revealed that patient mutations could cause loss of ion transport function in NHE9, resulting in altered endosomal trafficking and cell surface expression of receptors and neurotransmitter transporters. Contrasting with loss of function effects, a statistical analysis by Schwede et al. found upregulation of *SLC9A9*, along with downregulation of *SLC9A6* in autistic brains relative to non-autistic controls, emphasizing the importance of gene dosage in the autism phenotype (Schwede et al., 2014). *SLC9A9* was also one of the differentially expressed genes across multiple expression data sets that were within 10 cM of an autism-implicated locus. Genomic information for this study was collected from Autism Genetic Resource Exchange (AGRE) families (Vardarajan et al., 2013).
- **Attention deficit hyperactivity disorder (ADHD):** *SLC9A9* has been associated with ADHD, a heritable neuropsychiatric disorder. A genome wide scan of 126 affected sibling pairs identified a Quantitative Trait Locus (QTL) peak occurring at marker D3S1569, located in intron 5 of *SLC9A9* (Fisher et al., 2002). A year later, de Silva et al. published a clinical report showing ADHD and intellectual disability co-segregating with a pericentric inversion of chromosome 3 that disrupted *SLC9A9* along with *DOCK3* (de Silva et al., 2003). Genome wide association studies (GWAS) provide an opportunity to identify even non-classic disease risk genes that have a relatively milder effect in heterogenetic disorders. In a sample set of 958 parent-child trios, *SLC9A9* had the strongest overall association among the candidate genes they studied (Lasky-Su et al., 2008). Multiple association studies identified SNPs in *SLC9A9* to be significantly associated with ADHD and one study identified SNPs in six distinct regions of the gene. More recently, in a rat model of inattentive ADHD, age dependent abnormal expression of NHE9 and mutations that altered interaction with calcineurin homologous protein (CHP) were reported (Zhang-James et al., 2011).
- **Addiction:** Smoking initiation and current smoking are habits that have a genetic susceptibility component. In a network based GWAS study, *SLC9A9* stood out in a functional group with glutamate signaling, including the glutamate receptor and transporter (Vink et al., 2009). Furthermore, a family-based GWAS study identified significant associations between *SLC9A9* variants and alcohol-dependence phenotypes (Wang et al., 2013). The association of *SLC9A9* with inattention, smoking initiation, and alcohol dependence suggests a genetic connection between attention-deficit hyperactivity disorder and addictive behavior.
- **Alzheimer's Disease and Multiple Sclerosis:** eNHE may be potential risk factors common to both neurodevelopmental and neurodegenerative diseases. NHE6 has been implicated in Parkinsonism and deposition of tau neurofibrillary tangles (Garbern et al., 2010; Riess et al., 2013); similarly, emerging evidence links NHE9 with chronic neurodegenerative conditions, including Alzheimer's disease and multiple sclerosis. Significant association between NHE9 variants and Alzheimer's disease were identified in recent GWAS studies (Martinelli-Boneschi et al., 2013; Perez-Palma et al., 2014). Multiple sclerosis (MS) is an inflammatory demyelinating condition of the brain. A GWAS performed to compare responders vs. non-responders to interferon beta (IFNβ) treatment found significant association of an intronic NHE9 variant (rs9828519) with non-response to IFNβ therapy in MS patients (Esposito et al., 2013). In addition, polymorphisms within NHE9 have been reported in association with N-glycosylation variations, a common molecular mechanism in MS (Huffman et al., 2011; Mkhikian et al., 2011). Further studies are awaited to understand the link between NHE9 and multiple sclerosis. One possibility, based upon findings from model organisms and cultured cells, is that dysregulation of endosomal recycling of the IFN receptor could link NHE9 activity and response to IFNβ therapy.

In summary, clinical phenotypes resulting from genetic aberrations in *SLC9A6* and *SLC9A9* are distinct and diverse, despite overlapping endosomal cellular distributions of the two NHE isoforms. This indicates that NHE6 and NHE9 are non-redundant, and likely to differ in one or more of the following, including (i) brain expression patterns, (ii) precise subcellular distribution, (iii) transport function and regulation.

## Distribution of eNHE in the brain

**Figure 4:**
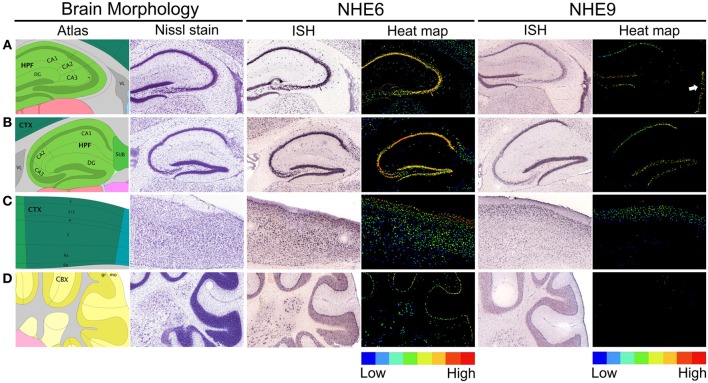
**Expression of eNHE in the hippocampus, cerebral cortex, and cerebellum. (A,B)** Brain morphology depicted using cartoon atlas and Nissl staining indicating coronal **(A)** and sagittal **(B)** planes of hippocampal formation (HPF), with the functionally distinct CA1, CA2, CA3, and dentate gyrus (DG) subfields. *In Situ* Hybridization (ISH) images of NHE6 and NHE9 expression in coronal **(A)** plane and sagittal **(B)** planes of hippocampus accompanied by false-color heat map adjacent to it, showing enriched eNHE expression in DG and CA1–CA3 (NHE6) and DG and CA1 (NHE9) (**A,B**, ISH and heat-map). NHE9 expression is also seen in the wall of lateral ventricle (VL) (**A**, ISH and white arrow in the heat-map). **(C)** Cartoon atlas and Nissl staining depicting cerebral cortex (CTX) in sagittal plane with labeled cortical layer boundaries. NHE9 is expressed in a gradient in the cortical layer subfield, in contrast to an NHE6 that is strongly expressed throughout the cortex (**C**, ISH and heat-map). **(D)** Cartoon atlas and Nissl staining showing cerebellar cortex (CBX) in sagittal plane. Prominent NHE6 expression is seen in the Purkinje cells arranged in a single layer between the molecular (mo) and granular (gr) layers. In contrast, NHE9 shows weak expression in the cerebellum (**D**, ISH and heat-map). False-color reference heat map (expression mask) scale indicating those cells that have the highest probability of gene expression (from low/blue to high/red). ISH expression data are from Allen Brain Atlas obtained from 56 day (8 weeks) old adult male C57BL/6J mice (available from: http://mouse.brain-map.org/) (Lein et al., 2007).

**Figure 5:**
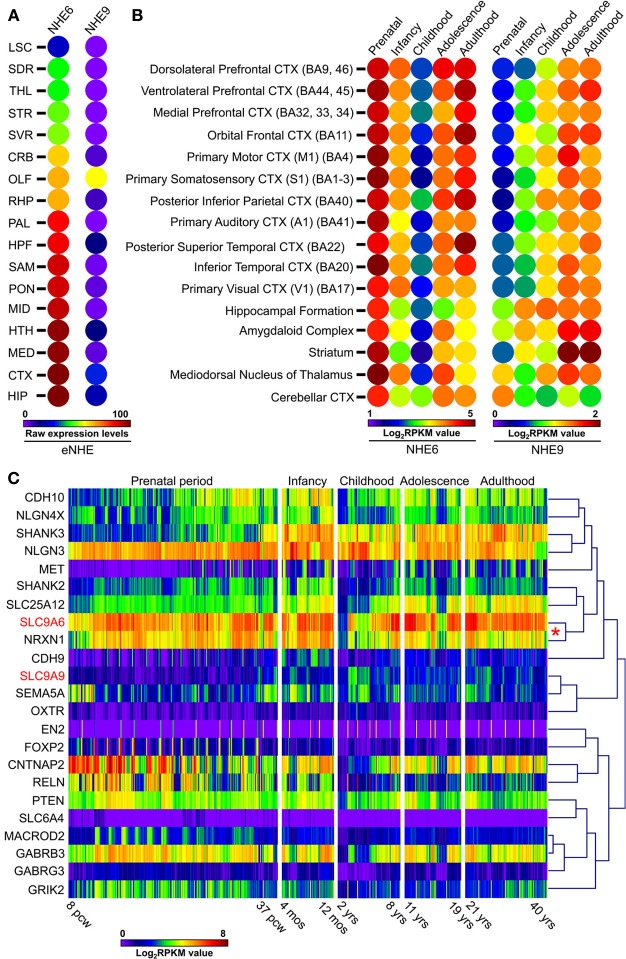
**Expression patterns of eNHE during normal brain development**. **(A)** Heat map of raw expression levels (from low/blue to high/red) of eNHE in various regions of the mouse brain determined from *in situ* hybridization data obtained from Allen Brain Atlas (http://mouse.brain-map.org/) (Lein et al., 2007). Genetically identical, inbred mice were used to limit individual differences and *in situ* data were normalized to a specially created reference atlas of mouse brain anatomy to control for differences between brain sections (Lein et al., 2007). Abbreviations: LSC, Lateral septal complex; SDR, Striatum dorsal region; THL, Thalamus; STR, Striatum; SVR, Striatum ventral region; CRB, Cerebellum; OLF, Olfactory bulb; RHP, Retrohippocampal region; PAL, Pallidum; HPF, Hippocampal formation; SAM, Striatum-like amygdalar nuclei; PON, Pons; MID, Midbrain; HTH, Hypothalamus; MED, Medulla; CTX, Cortex; HIP, Hippocampal region. **(B)** Heat map of average human NHE6 and NHE9 RNA-seq gene expression (from low/blue to high/red) plotted across different brain regions and different development stages which include: prenatal period (embryogenesis to birth), infancy (birth to 1 year), childhood (2–10 years), adolescence (11–20 years), and adulthood (21+ years). NHE6 shows highest expression prenatally during embryonic life, which lessens postnatally and again peaks during adulthood. In contrast, NHE9 expression is far lower embryonically, increases postnatally and peaks in the adulthood. Notably, a strong correlation was observed for NHE6 with NRXN1 a well-known autism candidate gene (red asterisk). **(C)** Hierarchical clustering and expression heat-map (from low/blue to high/red) of eNHE with 21 genes (Xu et al., 2012) with strong evidence for association with autism showing clustering of eNHE with many synapse associated autism genes during normal brain development. Notably, a strong correlation was observed for NHE6 with NRXN1 a well-known autism candidate gene (red asterisk). RNA-seq gene expression dataset included a total of 578 samples represented as log base 2 of RPKM values across different developmental periods and different brain regions (available from: http://www.brainspan.org) (Sunkin et al., 2013). Abbreviations: BA, Brodmann area; CTX, cortex; pcw, post-conception weeks; mos, postnatal months; yrs, age in years; RPKM, reads per kilobase of exon model per million mapped sequence reads.

The spatial and temporal regulation of eNHE expression in the brain may hold clues to their distinct and diverse phenotypes, including autism and neurological disorders. Indeed, NHE6 and NHE9 show distinct spatial (Figure 4) and temporal patterns of expression (Figure 5B). NHE6 is widely expressed in human brain and shows highest expression prenatally, decreases postnatally and again peaks during adulthood. On the other hand, NHE9 expression is far lower embryonically, increases postnatally to peak in adulthood (Figure 5B) (Sunkin et al., 2013). *In-situ* hybridization studies on the mouse brain indicate that the expression levels of NHE6 and NHE9 are high in the cortex relative to the other regions of the brain (Lein et al., 2007; Kondapalli et al., 2013), consistent with impairments in communication, social deficits, and cognitive function (Kanner, 1943; Howlin et al., 2000, 2004; Seltzer et al., 2004; Farley et al., 2009). Highest expression for NHE6 is observed in the hippocampus, which plays an important role in long-term memory (Figure 5A). The expression level of NHE9 in this region is next only to the olfactory lobe and cortex (Figure 5A). Although the significance of these expression patterns remains to be determined they are consistent with distinct and non-overlapping roles for the two eNHE isoforms. The robust expression levels of eNHE in areas potentially associated with ASD (Santangelo and Tsatsanis, 2005; Mitchell et al., 2009) may reflect the importance of ion and pH homeostasis on the normal functions of the cells in these regions of the brain.

Genetic architecture and co-expression networks may be important in understanding autism pathogenesis. To gather functional insights into the inherent patterns of co-expression between eNHE and other known ASD genes, we compared expression of eNHE with a “benchmark” gene set, consisting of 21 genes with strong evidence for ASD association, described previously (Xu et al., 2012). eNHE clusters with many synapse associated ASD genes during normal brain development (Figure 5C). Notably, a strong correlation was observed for NHE6 with NRXN1 a well-known autism candidate gene (Sudhof, 2008). Similar studies on postmortem autism brains also revealed altered eNHE expression and strong correlation with other synapse genes (Schwede et al., 2014). Furthermore, a strong induction of eNHE gene expression was observed during neuronal differentiation (Konopka et al., 2012). These observations strengthen the hypothesis that mechanisms regulating endosomal pH and eNHE activity may play an important role in brain development, neuronal differentiation, and synaptic function.

## Cellular model of endosomal NHE function at the synapse

As in non-neuronal cells, NHE6 and NHE9 show overlapping distribution to early and recycling endosomal compartments of neurons and astrocytes (Deane et al., 2013; Guterman, 2013; Kondapalli et al., 2013; Ouyang et al., 2013), suggesting a shared function in modulating ion homeostasis in these organelles. Deane et al. showed punctate distribution of NHE6 throughout the soma and dendrites of neurons that colocalizes with endosomal markers and the AMPA receptor subunit GluA1. Furthermore they showed enhanced recruitment of NHE6 to dendritic spines following NMDAR-dependent long-term potentiation, suggesting a role for NHE6 in learning and memory (Deane et al., 2013).

A viable knockout model of NHE6 is available from Jackson Labs (B6.129P2-Slc9a6^tm1Dgen^/J)^4^ with evidence of increased propensity for drug-induced seizures. The model was engineered by inserting the LacZ-Neo cassette into exon 6 of NHE6 that causes gene inactivation by introducing a stop codon and a polyadenylation termination signal. Strømme et al. first characterized this mouse model and showed that deletion of NHE6 results in dysfunction of endosomal lysosomal system and abnormal accumulation of GM2 ganglioside and unesterified cholesterol within late endosomes and lysosomes of neurons and progressive loss of neurons and cerebellar Purkinje cells. Behavioral tests showed motor hyperactivity, motor coordination deficits and evidences of cerebellar dysfunction in NHE6 knockout mice (Stromme et al., 2011). Ouyang et al. showed that NHE6 deletion results in hyperacidic endosomes in neurons and altered BDNF/TrkB signaling *in vitro* and proposed a role for NHE6 in neuronal arborization and circuit strength based on experiments in cultured neurons (Ouyang et al., 2013). Behavioral studies of mouse models are still forthcoming and may validate the link with autism, ADHD, and other neurological disorders.

No single neurobiological model currently explains ASD and related neurological disorders; however, those involving the synapse dominate cellular explanations. Neurons are morphologically, electrically, and chemically polarized cells. Tight regulation of development and connectivity of the synapse is central to normal brain function. Astrocytes play an important supporting role and have been implicated as critical players in syndromic autism and other neurodevelopmental disorders (Molofsky et al., 2012). The tripartite synapse includes the pre- and post-synaptic neuron, supported and regulated by the astrocyte. We present a model of eNHE function at all three components of the synapse. Although speculative, this model is constructed from currently known functions and a range of interesting, possible functions hypothesized based on published literature. These hypotheses are presented as a framework for future experimental testing and to spur more active research in this area.

### The presynaptic neuron

**Figure 6:**
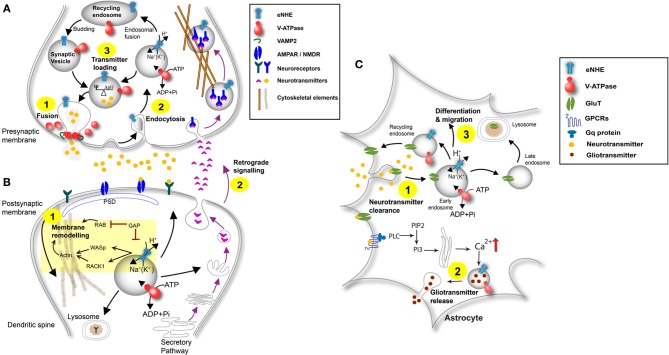
**Schematic of eNHE function at the synapse. (A) Presynaptic Neuron:** eNHE regulate luminal proton content to affect fusion of synaptic vesicles with the presynaptic membrane (*Step 1*). A role for eNHE in membrane assembly and endocytic retrieval of fused vesicles after the fused membrane undergoes lateral diffusion is depicted (*Step 2*). eNHE may influence the ratio between electrical and chemical gradients by dissipating ΔpH in favor of ΔΨ, thus controlling neurotransmitter loading (Goh et al., 2011) (*Step 3*). eNHE may also influence retrograde signaling in response to postsynaptic factors (*Step 2*; Figure 6B). **(B) Postsynaptic Neuron:** A model of eNHE function is presented in the context of membrane remodeling at dendritic spines (*Step 1*). Potential regulation of eNHE by Rab-GAP protein is extrapolated from the yeast ortholog, NHX1 and includes a possible interaction with Las17 ortholog WASP and the Gyp6 ortholog TBC1D5 (Ali et al., 2004; Kallay et al., 2011). Cytosolic domains of the eNHE bind scaffold protein RACK1, and the NHE6-Rack1 interaction controls receptor recycling in cultured cells (Ohgaki et al., 2008). RAB-mediated fusion and actin reorganization via WASP are required for membrane remodeling. Retrograde signaling occurs via factors secreted by the postsynaptic neuron that bind to receptors on the presynaptic membrane. A role of eNHE in retrograde signaling is shown (*Step 2;* see also Figure 6A). **(C) Astrocyte:** eNHE modulate luminal pH of endocytic and exocytic pathways in astrocytes (Kondapalli et al., 2013) to regulate vesicular trafficking, localization and turnover neurotransmitter transporters and neurotropic factor receptors (*Step 1*). Astrocytes surround synapses at which neurotransmitters are spilled over to stimulate astrocytic receptors leading to propagation of Ca^2+^ transients in astrocytes. Glial transmitters can be released from astrocytes in a Ca^2+^ dependent manner and can stimulate extra-synaptic receptors on adjacent neurons, leading to a dynamic modification of synaptic transmission. eNHE may differentially modulate the release of these gliotransmitters from the astrocyte (*Step 2*). Endosomal pH in astrocytes has been shown to affect cleavage of enzymes essential for Notch signaling, critical for astrocyte migration and differentiation (Valapala et al., 2013). A role for eNHE in these processes essential for migration and differentiation is depicted (*Step 3*).

Events at the presynaptic membrane are critically dependent on the regulated trafficking of neurotransmitter-loaded synaptic vesicles. The synaptic vesicle cycle is divided into discrete steps comprising fusion, retrieval and neurotransmitter loading, with potential roles for eNHE at each of these different stages (Figure 6A).

- **Synaptic vesicle fusion:** NHE6 has been reported in presynaptic terminal and in synaptic vesicles storing both GABA and glutamate, although its specific role remains to be investigated (Gronborg et al., 2010; Deane et al., 2013). Presynaptic vesicles make contact with the plasma membrane along the active zone where fusion of the vesicle membranes and exocytosis of the vesicle contents releases neurotransmitters (Sudhof, 2012) as depicted in *Step 1* (Figure 6). The ability of synaptic vesicles to dock and fuse at the active zone of the presynaptic terminal begins with a process called priming, in response to Ca^2+^ influx detected by synaptotagmins, and proceeds by pairing of SNARE proteins on the fusing membranes (Murthy and De Camilli, 2003). Within the vesicle lumen, the proton pumping V-ATPase acts in concert with chloride channels, Na^+^/K^+^-ATPase and eNHE to establish ionic conditions (Edwards, 2007; Goh et al., 2011). Together, they regulate vesicle pH, cation concentration and membrane potential. Recent evidence implicates a direct role for the membrane domain (V_o_) of the V-ATPase in synaptic membrane fusion, via a molecular interaction with the v-SNARE synaptobrevin (VAMP2) (Di Giovanni et al., 2010). Based on their findings, the authors speculate that optimal proton content is required for dissociation of the V_o_ domain from the V_1_ domain for interaction with VAMP2 (Morel et al., 2003; Di Giovanni et al., 2010). By extension, the eNHE could provide a leak pathway for protons and regulate luminal proton content to influence fusion of the synaptic vesicles with the membrane (*Step 1*, Figure 6A).
- **Synaptic vesicle endocytosis and trafficking:** The membrane of the fused vesicles undergoes lateral diffusion to a region outside the active zone where it is retrieved by endocytosis (Gundelfinger et al., 2003), depicted by *Step 2* (Figure 6A). The assembly of proteins in defined stoichiometry and their interaction with membrane lipids create microdomains that are required for assembling functional synaptic vesicles (Hannah et al., 1999). In polarized liver cells, regulation of endosomal pH by NHE6 directs sorting of membrane lipids to the apical surface for maintenance of cell polarity (Ohgaki et al., 2010). Similarly in polarized neuronal cells, eNHE may be critical in securing defined clusters of proteins and lipids at the synaptic membrane. There is evidence that eNHE themselves are routed to the endosomes by retrieval from the plasma membrane. Thus, in HeLa cells, a dominant negative mutant of dynamin reduces endosomal levels of NHE6 (Roxrud et al., 2009). Luminal pH is known to affect budding and coating of vesicles. In astrocytes, alkalinization of the endosomal lumen by NHE9 resulted in a significant increase of receptor-mediated endocytosis, as monitored by internalization of tagged transferrin (Kondapalli et al., 2013). One mechanism linking luminal pH to vesicle budding, again involves the V-ATPase: in kidney proximal tubules, endosomal V_o_ subunits transduce luminal pH signals to the vesicle assembling machinery by recruiting the small GTPase ARF6 and its cognate GTP/GDP exchange factor ARNO (Hurtado-Lorenzo et al., 2006). Similarly, the eNHE may also interact with trafficking machinery via their C-terminal cytoplasmic domains. Indeed, the yeast eNHE ortholog NHX1 interacts with, and is inhibited by the RAB-GTPase activating protein GYP6 to regulate luminal pH and vesicle trafficking (Ali et al., 2004). It is intriguing to note that phenotypes of null mutation in the δ subunit of AP3 clathrin adaptor protein involved in vesicle assembly and budding, overlap with those for eNHE, and include hyperactivity and epileptic EEG signatures in the hippocampus (Kantheti et al., 1998; Gilfillan et al., 2008). Mice with deletions in one of the AP3 subunits show defects specifically in the biogenesis of GABA containing synaptic vesicles (Kantheti et al., 1998). Similar defects in the inhibitory synapse could partly account for seizures comorbid in ASD patients, associated with mutations in NHE6 and NHE9 (Gilfillan et al., 2008; Morrow et al., 2008). These observations underscore the emerging role of the endosomal pathway at the synapse and in the pathology of neurodevelopmental disorders.
- **Neurotransmitter loading:** Newly endocytosed vesicles are transported back to the active zone after being filled with neurotransmitter, or translocated to a recycling endosome from which new vesicles bud and are filled with neurotransmitter on their way to the active zone (Gundelfinger et al., 2003; Sudhof, 2012) (*Step 3*; Figure 6A). Neurotransmitter uptake occurs through specific antiporters that harness one or both components of the transmembrane proton motive force: namely, the electrical (ΔΨ) and chemical (ΔpH) gradient (Goh et al., 2011). The uptake of monoamines and GABA is coupled to the pH gradient whereas glutamate uptake is coupled to a membrane potential. The unidirectional pumping of H^+^ by V-ATPase generates a membrane potential in the absence of charge compensation by other transporters and channels. However, the resulting ΔΨ is variably converted to a proton chemical gradient by the outward movement of positive charges (K^+^) or inward movement of negative ions (Cl^−^) via ion channels (Goh et al., 2011). Proton leak by eNHE tilts the ratio between electrical and chemical gradients by dissipating ΔpH in favor of ΔΨ, thus controlling neurotransmitter loading. Consistent with this, Goh et al. recently showed that monovalent Na^+^(K^+^)/H^+^ exchange activity promotes glutamate filling into synaptic vesicles by converting ΔpH into ΔΨ (Goh et al., 2011). Thus, the eNHE could regulate the content of synaptic vesicles to influence events at the presynaptic terminal.

### The postsynaptic neuron

Synaptic plasticity underlies cognition, learning, and memory. In the immature brain, altered synaptic signaling causes changes in neuronal connectivity, resulting in predisposition to neurological disorders. A model of eNHE function is presented in the context of membrane remodeling and retrograde signaling, two critical molecular steps at the postsynaptic membrane (Figure 6B).

(i) **Postsynaptic membrane remodeling at dendritic spines:** Signal processing at the synapse is mediated by an integration of excitatory and inhibitory inputs at the dendrites or the cell body of the postsynaptic neuron (Matsuzaki et al., 2004; Blanpied et al., 2008; Blanpied and Ehlers, 2011). An elaborate cluster of neurotransmitter-coupled receptors and ion channels, membrane trafficking proteins, cell adhesion molecules, signaling enzymes, cytoskeletal, and scaffolding proteins pack into the postsynaptic density (PSD) of “spines” that decorate the dendrites. A rich source of membrane bound organelles, including mitochondria, recycling endosomes, and smooth endoplasmic reticulum, underlies and supports the PSD. Spine morphology is modified based on neuronal activity and requires: (i) reorganization of the actin cytoskeleton and scaffold proteins to change shape and size, and (ii) movement of neurotransmitter receptors and ion channels on or off the postsynaptic membrane to change synaptic strength. It is worth noting that several NHE isoforms interact with the actin cytoskeleton and scaffold proteins through their cytoplasmic C-terminal domains (Paradiso et al., 2004; Cha and Donowitz, 2008; Donowitz et al., 2013) that range in size from ~150 (NHE6–9) to ~300 (NHE1–5) amino acids (*Step 1*; Figure 6B). Although specific information on binding partners of eNHE is scarce, it is intriguing that genetic variants in NHE9 linked to autism or ADHD have been mapped to this domain (de Silva et al., 2003; Morrow et al., 2008; Markunas et al., 2010; Zhang-James et al., 2011).

Endosomes in dendritic spines serve as readily accessible stores for membrane receptors, channels and membrane. Adding breadth to a promising area of research, an elegant study by Park et al. provided a mechanistic link between structural and functional plasticity (Park et al., 2004). Using pH sensitive cargo, they showed that upon receiving LTP inducing stimuli, recycling endosomes physically translocate to the spines where exocytosis of the cargo is promoted, resulting in the enlargement of dendritic spines. Inhibition of endosomal transport abolished the changes observed in spine morphology (Park et al., 2004). Consistent with a role in signal processing at the synapse, NHE6 was found at dendritic spines partially colocalizing with AMPAR subunit GluA1and exhibited enhanced translocation to dendritic spine heads during NMDR dependent long-term potentiation (Deane et al., 2013).

Garbern et al. identified a 9 base pair deletion in NHE6 in a patient family characterized by severe intellectual disability, ASD, and epilepsy. Postmortem samples of two males from the family revealed deposition of tau fibrils in neurons and glia. Tau phosphoproteins bind to microtubules and have been shown to promote their assembly and stability. Based on their observations and previously published studies the authors suggest that tau deposition maybe mediated by interaction with mutant NHE6. They speculate that interactions of eNHE with cytoskeletal elements may be critical for vesicular trafficking (Garbern et al., 2010).

(ii) **Retrograde signaling:** Release of neurotrophic signals and growth factors in an activity dependent manner by postsynaptic membranes drives synaptic maturation and growth through presynaptic endosomal signaling. This two-way transmission between pre- and post-synaptic membranes is essential for several processes including synapse formation, growth and global synaptic plasticity (da Silva and Wang, 2011). Genetic studies in rare familial cases indicate that abnormal synapse formation may be an underlying pathological pathway in autistic spectrum disorder (Arking et al., 2008; Lawson-Yuen et al., 2008; Pescosolido et al., 2012). Retrograde signaling occurs via factors secreted by the postsynaptic neuron that bind to receptors on the presynaptic membrane (*Step 2*; Figure 6B) (da Silva and Wang, 2011). The physical separation between soma and axon terminal requires the signal to travel long distances. Several lines of evidence now support a vesicular-based transport model with a “signaling endosome” capable of carrying the entire signaling complex into the soma where it recapitulates events that occurred at the axon terminal (Cosker et al., 2008) (Figure 6A). An interesting question concerns the factor(s) that regulate trafficking of this signaling endosome. How does the signaling endosome escape the recycling and lysosomal routes and enter the axonal transport pathway? Delcroix et al. showed that signaling endosomes of the Nerve growth factor (NGF) correspond to early endosomes (Delcroix et al., 2003), which was supported by Cui et al. who used quantum dots to track NGF retrograde signaling (Cui et al., 2007). Other studies suggest that RAB7-positive (late) endosomes and multivesicular bodies are associated with retrograde transport (Zerial and McBride, 2001; Rink et al., 2005; Deinhardt et al., 2006). Although we do not yet have a complete understanding of the endocytic pathway, one characteristic difference between these endosomes is their luminal pH (e.g., recycling ~6.5, late endosomes ~6.0) (Brett et al., 2006; Casey et al., 2010), known to be critically important for function (Brett et al., 2006; Casey et al., 2010). Knockdown or overexpression of eNHE significantly alters the pH of endosomes (Kondapalli et al., 2013; Ouyang et al., 2013). We, and others have shown that the change in pH arising from eNHE activity in the endosome results in altered cell surface expression of transferrin receptors and consequently transferrin uptake (Ohgaki et al., 2008; Kondapalli et al., 2013). Transferrin (Tfn) binds to cell surface receptors (TfR) at an extracellular pH of 7.4 with nanomolar binding affinity. Tfn-TfR complex clusters into clathrin coated pits to be delivered to early endosomes where the V-ATPase lowers the pH of endosomes to facilitate the release of iron from Tfn. Apo-Tfn remains bound to its receptor and is recycled back to the cell surface. Modulation of luminal pH by eNHE affects the release of iron from Tfn and consequently, recycling of internalized receptor to the surface. Similarly, we hypothesize that the eNHE may modulate dissociation of ligand-receptor dissociation in the signaling endosome, altering synaptic growth and neuronal connectivity to influence susceptibility to neuronal disorders.

### The astrocyte

The astrocyte, a type of glial cell, is an essential component of the tripartite synapse, modulating synaptic function for crucial determination of higher brain function and behavior (Smith, 1994; Araque et al., 1999). There is strong evidence that glial dysfunctions underlie psychiatric and brain disorders including autism, epilepsy, taupathies, and schizophrenia (Molofsky et al., 2012). Patients with NHE6 mutations present with prominent glial pathology (Garbern et al., 2010). We showed that both NHE6 and NHE9 are expressed in murine astrocytes, at levels slightly higher than those found in cortical neurons (Kondapalli et al., 2013). Importantly, manipulation of eNHE expression by knockdown or overexpression significantly altered endosomal pH (Kondapalli et al., 2013). Although most studies linking endosomal pH to astrocyte function have focused on the V-ATPase, we are only recently beginning to recognize the potential contribution of eNHE.

- **Neurotransmitter and neurotrophic factor clearance:** At the synapse, glial-specific ion channels and transporters clear K^+^ ions that accumulate during neuronal activity and remove neurotransmitters to terminate synaptic transmission. The neurotransmitter glutamate cannot be synthesized by neurons, which must therefore rely on the astrocyte to take up glutamate from the synaptic cleft, convert it into glutamine, which is then shuttled back into neurons as a renewable source of neurotransmitter. In murine astrocytes, elevated levels of NHE9 significantly alkalinized endosomal pH to increase cell surface expression and uptake activity of the glutamate transporter GLAST (Kondapalli et al., 2013). Conversely, it is proposed that autism-associated loss of function mutations in eNHE may reduce expression and activity of glutamate transporters at the synapse, consistent with elevated glutamate levels observed in patient brains, and resulting in the observed predisposition to seizures. Although, magnetic resonance spectroscopy (MRS) signal abnormality from a clinical study in a single patient could be open to a variety of different interpretations other than problems in glutamate within the synapse, there are emerging studies that support a role for Na^+^(K^+^)/H^+^ exchange in glutamatergic transmission (Vink et al., 2009; Goh et al., 2011; Kondapalli et al., 2013). It has been shown that astrocytes affect synaptic plasticity by regulating the extracellular availability of brain-derived neurotrophic factor (BDNF). Neurons synthesize BDNF and release it in the precursor form (pro-BDNF), which can then be processed in the extracellular space by plasmin. Pro-BDNF promotes long-term depression whereas the mature form induces long-term potentiation. As both these forms have opposite effects on synaptic transmission, it is crucial to regulate their availability in the extracellular space. Pro-BDNF binds to p75 receptors on the astrocyte surface and is rapidly endocytosed. The mechanisms regulating the recycling and release of neurotransmitters and neurotropic factors are poorly understood. The eNHE could potentially regulate these processes by controlling pH within the lumen of endocytic and exocytic pathways in astrocytes to regulate vesicular trafficking, localization and turnover of critical components of synaptic function, including neurotransmitter transporters and neurotropic factor receptors (*Step 1*; Figure 6C).
- **Gliotransmitter release:** Ca^2+^ oscillations in astrocytes induce release of gliotransmitters (e.g., glutamate, D-serine, ATP, tumor necrosis factor) that in turn can elicit neuronal synchronization, synaptic modulation and plasticity (e.g., by controlling insertion of AMPA receptors or regulating NMDA receptor function) (Parpura and Zorec, 2010). Because glutamate uptake into synaptic vesicles is mediated by VGLUT transporters that couple to the vesicular proton gradient, alkalinizing endosomal pH using the V-ATPase inhibitor bafilomycin diminished glutamate release from astrocytes, without suppressing Ca^2+^ elevations (Montana et al., 2004). Similarly, sequestration of D-serine into glial vesicles was dependent on ΔpH and ΔΨ as evidenced by sensitivity to both nigericin (a K^+^/H^+^ exchanger) and valinomycin (a K^+^ ionophore) that dissipate pH and membrane potential gradients respectively (Martineau et al., 2013). On the other hand, V-ATPase inhibitors potently stimulated exocytic release of the glial cell-derived neurotrophic factor GDNF from a variety of glial cell lines (Nishiguchi et al., 2003). Thus, endosomal pH is likely to differentially modulate release of various gliotransmitters and growth factors from the astrocyte (*Step 2*; Figure 6C).
- **Astrocyte differentiation and migration:** Notch signaling, critical for astrocyte migration and differentiation, requires cleavage by the γ-secretase enzyme within acidic endo-lysosomal compartments. Recently, impairment of endo-lysosomal acidification was shown to underlie defective Notch processing and signaling in optic nerve astrocytes in the Nuc1 mutant rat (Valapala et al., 2013). Although the mechanism underlying the loss of acidification is not known, the authors speculate a dysfunction of the V-ATPase. However, alterations in eNHE activity may also underlie defects in endo-lysosomal pH and impact astrocyte differentiation and migration (*Step 3*; Figure 6C).

## Conclusions and prospectives

In summary, endosomal pH and membrane potential are set by a balance of inward pumping of protons by V-ATPase and outward leak via eNHE. Although the role of the ubiquitous H^+^ pumping V-ATPase has long been appreciated from previous studies, this review highlights how eNHE activity is critical in setting luminal pH and ion content by providing a leak pathway for protons. Future studies investigating vesicular trafficking and transport on synapse function, neuronal plasticity or differentiation should take into account eNHE function. Finally, safe and effective therapies for the treatment of autism and related disorders remain a pressing need. It should be possible to counter the effect of altering eNHE function by using drugs that act as weak acids or bases to lower or raise the pH of endo-lysosomal compartments, respectively. Since membrane transporters are highly “druggable” targets with proven success in the clinic, a search for selective inhibitors or potentiators of eNHE activity should be an immediate priority. Inhibitors against better-known plasma membrane NHE isoforms could also be screened for efficacy against eNHE using *in silico* (Faraone and Zhang-James, 2013), *in vitro*, and *in vivo* approaches.

## Author contributions

Kalyan C. Kondapalli, Hari Prasad, and Rajini Rao wrote and prepared this article. All authors have read and approved this manuscript.

### Conflict of interest statement

The authors declare that the research was conducted in the absence of any commercial or financial relationships that could be construed as a potential conflict of interest.

## Footnotes

^1^OMIM 300243. *Online Mendelian Inheritance in Man, OMIM*®. Available online at: http://www.omim.org/entry/300243

^2^National Institute of Mental Health. *Autism Spectrum Disorders (Pervasive Developmental Disorders)*. Available online at: http://www.nimh.nih.gov/health/publications/autism/index.shtml [Accessed 03/07/2014].

^3^OMIM 613410. *Online Mendelian Inheritance in Man, OMIM*®. Available online at: http://www.omim.org/entry/613410

^4^B6.129P2-Slc9a6^tm1Dgen^/*J. The Jackson Laboratory*. Available online at: http://jaxmice.jax.org/strain/005843.html

## Abbreviations

NHE: sodium proton exchanger
SLC: solute carrier
ASD: autism spectrum disorders
MRS: magnetic resonance spectroscopy
eNHE: endosomal sodium hydrogen exchanger
XLID: X-linked intellectual disability
ADHD: attention deficit hyperactivity disorder.

## References

[B1] Albrecht U. Sutcliffe J. S. Cattanach B. M. Beechey C. V. Armstrong D. Eichele G. (1997). Imprinted expression of the murine Angelman syndrome gene, Ube3a, in hippocampal and Purkinje neurons. Nat. Genet. 17, 75–78 10.1038/ng0997-75 9288101

[B2] Ali R. Brett C. L. Mukherjee S. Rao R. (2004). Inhibition of sodium/proton exchange by a Rab-GTPase-activating protein regulates endosomal traffic in yeast. J. Biol. Chem. 279, 4498–4506 10.1074/jbc.M307446200 14610088

[B3] Amr M. Raddad D. El-Mehesh F. Bakr A. Sallam K. Amin T. (2012). Comorbid psychiatric disorders in Arab children with autism spectrum disorders. Res. Autism Spectr. Disord. 6, 240–248 10.1016/j.rasd.2011.05.005

[B4] Andres Z. Perez-Hormaeche J. Leidi E. O. Schlucking K. Steinhorst L. Mclachlan D. H. (2014). Control of vacuolar dynamics and regulation of stomatal aperture by tonoplast potassium uptake. Proc. Natl. Acad. Sci. U.S.A. 111, E1806–E1814 10.1073/pnas.1320421111 24733919 PMC4035970

[B5] Antshel K. M. Zhang-James Y. Faraone S. V. (2013). The comorbidity of ADHD and autism spectrum disorder. Expert Rev. Neurother. 13, 1117–1128 10.1586/14737175.2013.840417 24117274

[B6] Apse M. P. Aharon G. S. Snedden W. A. Blumwald E. (1999). Salt tolerance conferred by overexpression of a vacuolar Na^+^/H^+^ antiport in Arabidopsis. Science 285, 1256–1258 10.1126/science.285.5431.1256 10455050

[B7] Araque A. Parpura V. Sanzgiri R. P. Haydon P. G. (1999). Tripartite synapses: glia, the unacknowledged partner. Trends Neurosci. 22, 208–215 10.1016/S0166-2236(98)01349-6 10322493

[B8] Arking D. E. Cutler D. J. Brune C. W. Teslovich T. M. West K. Ikeda M. (2008). A common genetic variant in the neurexin superfamily member CNTNAP2 increases familial risk of autism. Am. J. Hum. Genet. 82, 160–164 10.1016/j.ajhg.2007.09.015 18179894 PMC2253968

[B9] Ashkenazy H. Erez E. Martz E. Pupko T. Ben-Tal N. (2010). ConSurf 2010: calculating evolutionary conservation in sequence and structure of proteins and nucleic acids. Nucleic Acids Res. 38(Suppl.), W529–W533 10.1093/nar/gkq399 20478830 PMC2896094

[B10] Baio J. (2014). Prevalence of autism spectrum disorder among children aged 8 years—autism and developmental disabilities monitoring network, 11 sites, United States, 2010. MMWR. Surveill. Summ. 63(Suppl. 2), 1–21 Available online at: http://www.cdc.gov/mmwr/preview/mmwrhtml/ss6302a1.htm 24670961

[B11] Ben-David E. Granot-Hershkovitz E. Monderer-Rothkoff G. Lerer E. Levi S. Yaari M. (2011). Identification of a functional rare variant in autism using genome-wide screen for monoallelic expression. Hum. Mol. Genet. 20, 3632–3641 10.1093/hmg/ddr283 21680558

[B12] Blanpied T. A. Ehlers M. D. (2011). Membrane trafficking and cytoskeletal dynamics in neuronal function. Mol. Cell. Neurosci. 48, 267–268 10.1016/j.mcn.2011.09.006 22035734

[B13] Blanpied T. A. Kerr J. M. Ehlers M. D. (2008). Structural plasticity with preserved topology in the postsynaptic protein network. Proc. Natl. Acad. Sci. U.S.A. 105, 12587–12592 10.1073/pnas.0711669105 18723686 PMC2519044

[B14] Bosemani T. Zanni G. Hartman A. L. Cohen R. Huisman T. A. Bertini E. (2013). Christianson syndrome: spectrum of neuroimaging findings. Neuropediatrics. [Epub ahead of print]. 10.1055/s-0033-1363091 24285247

[B15] Brett C. L. Donowitz M. Rao R. (2005a). Evolutionary origins of eukaryotic sodium/proton exchangers. Am. J. Physiol. Cell Physiol. 288, C223–C239 10.1152/ajpcell.00360.2004 15643048

[B16] Brett C. L. Donowitz M. Rao R. (2006). Does the proteome encode organellar pH? FEBS Lett. 580, 717–719 10.1016/j.febslet.2005.12.103 16413548

[B17] Brett C. L. Kallay L. Hua Z. Green R. Chyou A. Zhang Y. (2011). Genome-wide analysis reveals the vacuolar pH-stat of *Saccharomyces cerevisiae*. PLoS ONE 6:e17619 10.1371/journal.pone.0017619 21423800 PMC3056714

[B18] Brett C. L. Tukaye D. N. Mukherjee S. Rao R. (2005b). The yeast endosomal Na^+^K^+^/H^+^ exchanger Nhx1 regulates cellular pH to control vesicle trafficking. Mol. Biol. Cell 16, 1396–1405 10.1091/mbc.E04-11-0999 15635088 PMC551501

[B19] Brett C. L. Wei Y. Donowitz M. Rao R. (2002). Human Na(+)/H(+) exchanger isoform 6 is found in recycling endosomes of cells, not in mitochondria. Am. J. Physiol. Cell Physiol. 282, C1031–C1041 10.1152/ajpcell.00420.2001 11940519

[B19a] Brookes K. Xu X. Chen W. Zhou K. Neale B. Lowe N. (2006). The analysis of 51 genes in DSM-IV combined type attention deficit hyperactivity disorder: association signals in DRD4, DAT1 and 16 other genes. Mol. Psychiatry 11, 934–953 10.1038/sj.mp.4001869 16894395

[B20] Canitano R. (2007). Epilepsy in autism spectrum disorders. Eur. Child Adolesc. Psychiatry 16, 61–66 10.1007/s00787-006-0563-2 16932856

[B21] Casey J. R. Grinstein S. Orlowski J. (2010). Sensors and regulators of intracellular pH. Nat. Rev. Mol. Cell Biol. 11, 50–61 10.1038/nrm2820 19997129

[B22] Cha B. Donowitz M. (2008). The epithelial brush border Na^+^/H^+^ exchanger NHE3 associates with the actin cytoskeleton by binding to ezrin directly and via PDZ domain-containing Na^+^/H^+^ exchanger regulatory factor (NHERF) proteins. Clin. Exp. Pharmacol. Physiol. 35, 863–871 10.1111/j.1440-1681.2008.04931.x 18430067

[B23] Chahrour M. Jung S. Y. Shaw C. Zhou X. Wong S. T. Qin J. (2008). MeCP2, a key contributor to neurological disease, activates and represses transcription. Science 320, 1224–1229 10.1126/science.1153252 18511691 PMC2443785

[B24] Christianson A. L. Stevenson R. E. van der Meyden C. H. Pelser J. Theron F. W. Van Rensburg P. L. (1999). X linked severe mental retardation, craniofacial dysmorphology, epilepsy, ophthalmoplegia, and cerebellar atrophy in a large South African kindred is localised to Xq24-q27. J. Med. Genet. 36, 759–766 10.1136/jmg.36.10.759 10528855 PMC1734236

[B25] Cosker K. E. Courchesne S. L. Segal R. A. (2008). Action in the axon: generation and transport of signaling endosomes. Curr. Opin. Neurobiol. 18, 270–275 10.1016/j.conb.2008.08.005 18778772 PMC2693191

[B26] Cui B. Wu C. Chen L. Ramirez A. Bearer E. L. Li W. P. (2007). One at a time, live tracking of NGF axonal transport using quantum dots. Proc. Natl. Acad. Sci. U.S.A. 104, 13666–13671 10.1073/pnas.0706192104 17698956 PMC1959439

[B27] da Silva S. Wang F. (2011). Retrograde neural circuit specification by target-derived neurotrophins and growth factors. Curr. Opin. Neurobiol. 21, 61–67 10.1016/j.conb.2010.07.007 20810276 PMC3008758

[B28] Deane E. C. Ilie A. E. Sizdahkhani S. Das Gupta M. Orlowski J. McKinney R. A. (2013). Enhanced recruitment of endosomal Na^+^/H^+^ exchanger NHE6 into dendritic spines of hippocampal pyramidal neurons during NMDA receptor-dependent long-term potentiation. J. Neurosci. 33, 595–610 10.1523/JNEUROSCI.2583-12.2013 23303939 PMC6704919

[B29] Deinhardt K. Salinas S. Verastegui C. Watson R. Worth D. Hanrahan S. (2006). Rab5 and Rab7 control endocytic sorting along the axonal retrograde transport pathway. Neuron 52, 293–305 10.1016/j.neuron.2006.08.018 17046692

[B30] Delcroix J. D. Valletta J. S. Wu C. Hunt S. J. Kowal A. S. Mobley W. C. (2003). NGF signaling in sensory neurons: evidence that early endosomes carry NGF retrograde signals. Neuron 39, 69–84 10.1016/S0896-6273(03)00397-0 12848933

[B31] Delorme R. Ey E. Toro R. Leboyer M. Gillberg C. Bourgeron T. (2013). Progress toward treatments for synaptic defects in autism. Nat. Med. 19, 685–694 10.1038/nm.3193 23744158

[B32] de Silva M. G. Elliott K. Dahl H. H. Fitzpatrick E. Wilcox S. Delatycki M. (2003). Disruption of a novel member of a sodium/hydrogen exchanger family and DOCK3 is associated with an attention deficit hyperactivity disorder-like phenotype. J. Med. Genet. 40, 733–740 10.1136/jmg.40.10.733 14569117 PMC1735283

[B33] Devlin B. Scherer S. W. (2012). Genetic architecture in autism spectrum disorder. Curr. Opin. Genet. Dev. 22, 229–237 10.1016/j.gde.2012.03.002 22463983

[B34] Di Giovanni J. Boudkkazi S. Mochida S. Bialowas A. Samari N. Leveque C. (2010). V-ATPase membrane sector associates with synaptobrevin to modulate neurotransmitter release. Neuron 67, 268–279 10.1016/j.neuron.2010.06.024 20670834

[B35] Donowitz M. Ming Tse C. Fuster D. (2013). SLC9/NHE gene family, a plasma membrane and organellar family of Na(+)/H(+) exchangers. Mol. Aspects Med. 34, 236–251 10.1016/j.mam.2012.05.001 23506868 PMC3724465

[B36] Edwards R. H. (2007). The neurotransmitter cycle and quantal size. Neuron 55, 835–858 10.1016/j.neuron.2007.09.001 17880890

[B37] Esposito F. Sorosina M. Lim E. Brambilla P. Romeo M. Rodegher M. (2013). An SLC9A9 variant influences treatment response in interferon beta treated multiple sclerosis patients. Neurology 80, 1 Available online at: http://www.neurology.org/cgi/content/meeting_abstract/80/1_MeetingAbstracts/P05.141

[B38] Faraone S. V. Zhang-James Y. (2013). Can sodium/hydrogen exchange inhibitors be repositioned for treating attention deficit hyperactivity disorder? An *in silico* approach. Am. J. Med. Genet. B Neuropsychiatr. Genet. 162, 711–717 10.1002/ajmg.b.32155 24132903

[B39] Farley M. A. McMahon W. M. Fombonne E. Jenson W. R. Miller J. Gardner M. (2009). Twenty-year outcome for individuals with autism and average or near-average cognitive abilities. Autism Res. 2, 109–118 10.1002/aur.69 19455645

[B40] Fichou Y. Bahi-Buisson N. Nectoux J. Chelly J. Heron D. Cuisset L. (2009). Mutation in the SLC9A6 gene is not a frequent cause of sporadic Angelman-like syndrome. Eur. J. Hum. Genet. 17, 1378–1380 10.1038/ejhg.2009.82 19471312 PMC2986687

[B41] Fisher S. E. Francks C. McCracken J. T. McGough J. J. Marlow A. J. MacPhie I. L. (2002). A genomewide scan for loci involved in attention-deficit/hyperactivity disorder. Am. J. Hum. Genet. 70, 1183–1196 10.1086/340112 11923911 PMC447594

[B42] Flavell S. W. Greenberg M. E. (2008). Signaling mechanisms linking neuronal activity to gene expression and plasticity of the nervous system. Annu. Rev. Neurosci. 31, 563–590 10.1146/annurev.neuro.31.060407.125631 18558867 PMC2728073

[B43] Flavell S. W. Kim T. K. Gray J. M. Harmin D. A. Hemberg M. Hong E. J. (2008). Genome-wide analysis of MEF2 transcriptional program reveals synaptic target genes and neuronal activity-dependent polyadenylation site selection. Neuron 60, 1022–1038 10.1016/j.neuron.2008.11.029 19109909 PMC2630178

[B44] Folstein S. E. Rosen-Sheidley B. (2001). Genetics of autism: complex aetiology for a heterogeneous disorder. Nat. Rev. Genet. 2, 943–955 10.1038/35103559 11733747

[B45] Fombonne E. (2003). Epidemiological surveys of autism and other pervasive developmental disorders: an update. J. Autism Dev. Disord. 33, 365–382 10.1023/A:1025054610557 12959416

[B46] Fukada-Tanaka S. Inagaki Y. Yamaguchi T. Saito N. Iida S. (2000). Colour-enhancing protein in blue petals. Nature 407, 581 10.1038/35036683 11034195

[B47] Garbern J. Y. Neumann M. Trojanowski J. Q. Lee V. M. Feldman G. Norris J. W. (2010). A mutation affecting the sodium/proton exchanger, SLC9A6, causes mental retardation with tau deposition. Brain 133, 1391–1402 10.1093/brain/awq071 20395263 PMC2859154

[B48] Gilfillan G. D. Selmer K. K. Roxrud I. Smith R. Kyllerman M. Eiklid K. (2008). SLC9A6 mutations cause X-linked mental retardation, microcephaly, epilepsy, and ataxia, a phenotype mimicking Angelman syndrome. Am. J. Hum. Genet. 82, 1003–1010 10.1016/j.ajhg.2008.01.013 18342287 PMC2427207

[B49] Gjevik E. Eldevik S. Fjaeran-Granum T. Sponheim E. (2011). Kiddie-SADS reveals high rates of DSM-IV disorders in children and adolescents with autism spectrum disorders. J. Autism Dev. Disord. 41, 761–769 10.1007/s10803-010-1095-7 20824493 PMC3094530

[B50] Goh G. Y. Huang H. Ullman J. Borre L. Hnasko T. S. Trussell L. O. (2011). Presynaptic regulation of quantal size: K^+^/H^+^ exchange stimulates vesicular glutamate transport. Nat. Neurosci. 14, 1285–1292 10.1038/nn.2898 21874016 PMC3183113

[B51] Gronborg M. Pavlos N. J. Brunk I. Chua J. J. Munster-Wandowski A. Riedel D. (2010). Quantitative comparison of glutamatergic and GABAergic synaptic vesicles unveils selectivity for few proteins including MAL2, a novel synaptic vesicle protein. J. Neurosci. 30, 2–12 10.1523/JNEUROSCI.4074-09.2010 20053882 PMC6632534

[B52] Gundelfinger E. D. Kessels M. M. Qualmann B. (2003). Temporal and spatial coordination of exocytosis and endocytosis. Nat. Rev. Mol. Cell Biol. 4, 127–139 10.1038/nrm1016 12563290

[B53] Guterman M. (2013). NHE6 and NHE9 are Sodium Hydrogen Exchangers Found on Separate Mobile Endosome Populations in Neuronal Dendrites. Masters thesis, Concordia University, Montreal, QC

[B54] Han W. Kim K. H. Jo M. J. Lee J. H. Yang J. Doctor R. B. (2006). Shank2 associates with and regulates Na^+^/H^+^ exchanger 3. J. Biol. Chem. 281, 1461–1469 10.1074/jbc.M509786200 16293618

[B55] Hannah M. J. Schmidt A. A. Huttner W. B. (1999). Synaptic vesicle biogenesis. Annu. Rev. Cell Dev. Biol. 15, 733–798 10.1146/annurev.cellbio.15.1.733 10611977

[B56] Hill J. K. Brett C. L. Chyou A. Kallay L. M. Sakaguchi M. Rao R. (2006). Vestibular hair bundles control pH with (Na^+^, K^+^)/H^+^ exchangers NHE6 and NHE9. J. Neurosci. 26, 9944–9955 10.1523/JNEUROSCI.2990-06.2006 17005858 PMC6674470

[B57] Howlin P. Goode S. Hutton J. Rutter M. (2004). Adult outcome for children with autism. J. Child Psychol. Psychiatry 45, 212–229 10.1111/j.1469-7610.2004.00215.x 14982237

[B58] Howlin P. Mawhood L. Rutter M. (2000). Autism and developmental receptive language disorder–a follow-up comparison in early adult life. II: social, behavioural, and psychiatric outcomes. J. Child Psychol. Psychiatry 41, 561–578 10.1111/1469-7610.00643 10946749

[B59] Huffman J. E. Knezevic A. Vitart V. Kattla J. Adamczyk B. Novokmet M. (2011). Polymorphisms in B3GAT1, SLC9A9 and MGAT5 are associated with variation within the human plasma N-glycome of 3533 European adults. Hum. Mol. Genet. 20, 5000–5011 10.1093/hmg/ddr414 21908519

[B60] Hunte C. Screpanti E. Venturi M. Rimon A. Padan E. Michel H. (2005). Structure of a Na^+^/H^+^ antiporter and insights into mechanism of action and regulation by pH. Nature 435, 1197–1202 10.1038/nature03692 15988517

[B61] Hurtado-Lorenzo A. Skinner M. El Annan J. Futai M. Sun-Wada G. H. Bourgoin S. (2006). V-ATPase interacts with ARNO and Arf6 in early endosomes and regulates the protein degradative pathway. Nat. Cell Biol. 8, 124–136 10.1038/ncb1348 16415858

[B61a] Hu H. Wrogemann K. Kalscheuer V. Tzschach A. Richard H. Haas S. A. (2009). Mutation screening in 86 known X-linked mental retardation genes by droplet-based multiplex PCR and massive parallel sequencing. Hugo. J. 3, 41–49 10.1007/s11568-010-9137-y 21836662 PMC2882650

[B62] Ilie A. Weinstein E. Boucher A. McKinney R. A. Orlowski J. (2013). Impaired posttranslational processing and trafficking of an endosomal Na/H exchanger NHE6 mutant (DeltaWST) associated with X-linked intellectual disability and autism. Neurochem. Int. . [Epub ahead of print]. 10.1016/j.neuint.2013.09.020 24090639

[B63] Iossifov I. Ronemus M. Levy D. Wang Z. Hakker I. Rosenbaum J. (2012). *De novo* gene disruptions in children on the autistic spectrum. Neuron 74, 285–299 10.1016/j.neuron.2012.04.009 22542183 PMC3619976

[B64] Jozwiak S. Kotulska K. Domanska-Pakiela D. Lojszczyk B. Syczewska M. Chmielewski D. (2011). Antiepileptic treatment before the onset of seizures reduces epilepsy severity and risk of mental retardation in infants with tuberous sclerosis complex. Eur. J. Paediatr. Neurol. 15, 424–431 10.1016/j.ejpn.2011.03.010 21507691

[B65] Kallay L. M. Brett C. L. Tukaye D. N. Wemmer M. A. Chyou A. Odorizzi G. (2011). Endosomal Na^+^ (K^+^)/H^+^ exchanger Nhx1/Vps44 functions independently and downstream of multivesicular body formation. J. Biol. Chem. 286, 44067–44077 10.1074/jbc.M111.282319 21998311 PMC3243563

[B66] Kanner L. (1943). Autistic disturbances of affective contact. Nervous Child 2, 217–250 4880460

[B67] Kantheti P. Qiao X. Diaz M. E. Peden A. A. Meyer G. E. Carskadon S. L. (1998). Mutation in AP-3 delta in the mocha mouse links endosomal transport to storage deficiency in platelets, melanosomes, and synaptic vesicles. Neuron 21, 111–122 10.1016/S0896-6273(00)80519-X 9697856

[B68] Kondapalli K. C. Hack A. Schushan M. Landau M. Ben-Tal N. Rao R. (2013). Functional evaluation of autism-associated mutations in NHE9. Nat. Commun. 4, 2510 10.1038/ncomms3510 24065030 PMC3815575

[B69] Konopka G. Wexler E. Rosen E. Mukamel Z. Osborn G. E. Chen L. (2012). Modeling the functional genomics of autism using human neurons. Mol. Psychiatry 17, 202–214 10.1038/mp.2011.60 21647150 PMC3170664

[B70] Landau M. Herz K. Padan E. Ben-Tal N. (2007). Model structure of the Na^+^/H^+^ exchanger 1 (NHE1): functional and clinical implications. J. Biol. Chem. 282, 37854–37863 10.1074/jbc.M705460200 17981808

[B71] Landrigan P. J. (2010). What causes autism? Exploring the environmental contribution. Curr. Opin. Pediatr. 22, 219–225 10.1097/MOP.0b013e328336eb9a 20087185

[B72] Lasky-Su J. Neale B. M. Franke B. Anney R. J. Zhou K. Maller J. B. (2008). Genome-wide association scan of quantitative traits for attention deficit hyperactivity disorder identifies novel associations and confirms candidate gene associations. Am. J. Med. Genet. B Neuropsychiatr. Genet. 147B, 1345–1354 10.1002/ajmg.b.30867 18821565

[B73] Lawson-Yuen A. Saldivar J. S. Sommer S. Picker J. (2008). Familial deletion within NLGN4 associated with autism and Tourette syndrome. Eur. J. Hum. Genet. 16, 614–618 10.1038/sj.ejhg.5202006 18231125

[B74] Lee C. Kang H. J. von Ballmoos C. Newstead S. Uzdavinys P. Dotson D. L. (2013). A two-domain elevator mechanism for sodium/proton antiport. Nature 501, 573–577 10.1038/nature12484 23995679 PMC3914025

[B75] Lein E. S. Hawrylycz M. J. Ao N. Ayres M. Bensinger A. Bernard A. (2007). Genome-wide atlas of gene expression in the adult mouse brain. Nature 445, 168–176 10.1038/nature05453 17151600

[B76] Lin X. Barber D. L. (1996). A calcineurin homologous protein inhibits GTPase-stimulated Na-H exchange. Proc. Natl. Acad. Sci. U.S.A. 93, 12631–12636 10.1073/pnas.93.22.12631 8901634 PMC38044

[B77] Liu X. Q. Paterson A. D. Szatmari P. (2008). Genome-wide linkage analyses of quantitative and categorical autism subphenotypes. Biol. Psychiatry 64, 561–570 10.1016/j.biopsych.2008.05.023 18632090 PMC2670970

[B78] Madrigal I. Fernandez-Burriel M. Rodriguez-Revenga L. Cabrera J. C. Marti M. Mur A. (2010). Xq26.2-q26.3 microduplication in two brothers with intellectual disabilities: clinical and molecular characterization. J. Hum. Genet. 55, 822–826 10.1038/jhg.2010.119 20861843

[B79] Markunas C. A. Quinn K. S. Collins A. L. Garrett M. E. Lachiewicz A. M. Sommer J. L. (2010). Genetic variants in SLC9A9 are associated with measures of attention-deficit/hyperactivity disorder symptoms in families. Psychiatr. Genet. 20, 73–81 10.1097/YPG.0b013e3283351209 20032819 PMC3085270

[B80] Martineau M. Shi T. Puyal J. Knolhoff A. M. Dulong J. Gasnier B. (2013). Storage and uptake of D-serine into astrocytic synaptic-like vesicles specify gliotransmission. J. Neurosci. 33, 3413–3423 10.1523/JNEUROSCI.3497-12.2013 23426669 PMC3772647

[B81] Martinelli-Boneschi F. Giacalone G. Magnani G. Biella G. Coppi E. Santangelo R. (2013). Pharmacogenomics in Alzheimer's disease: a genome-wide association study of response to cholinesterase inhibitors. Neurobiol. Aging 34, 1711.e7–1711.e13 10.1016/j.neurobiolaging.2012.12.008 23374588

[B82] Matsuzaki M. Honkura N. Ellis-Davies G. C. Kasai H. (2004). Structural basis of long-term potentiation in single dendritic spines. Nature 429, 761–766 10.1038/nature02617 15190253 PMC4158816

[B83] Mellman I. (1992). The importance of being acid: the role of acidification in intracellular membrane traffic. J. Exp. Biol. 172, 39–45 1491231 10.1242/jeb.172.1.39

[B83a] Mick E. Todorov A. Smalley S. Hu X. Loo S. Todd R. D. (2010). Family-based genome-wide association scan of attention-deficit/hyperactivity disorder. J. Am. Acad. Child. Adolesc. Psychiatry. 49, 898–905e3. 10.1016/j.jaac.2010.02.014 20732626 PMC3730251

[B84] Mignot C. Heron D. Bursztyn J. Momtchilova M. Mayer M. Whalen S. (2013). Novel mutation in SLC9A6 gene in a patient with Christianson syndrome and retinitis pigmentosum. Brain Dev. 35, 172–176 10.1016/j.braindev.2012.03.010 22541666

[B85] Mitchell S. R. Reiss A. L. Tatusko D. H. Ikuta I. Kazmerski D. B. Botti J. A. (2009). Neuroanatomic alterations and social and communication deficits in monozygotic twins discordant for autism disorder. Am. J. Psychiatry 166, 917–925 10.1176/appi.ajp.2009.08101538 19605538

[B86] Mkhikian H. Grigorian A. Li C. F. Chen H. L. Newton B. Zhou R. W. (2011). Genetics and the environment converge to dysregulate N-glycosylation in multiple sclerosis. Nat. Commun. 2, 334 10.1038/ncomms1333 21629267 PMC3133923

[B87] Molofsky A. V. Krencik R. Ullian E. M. Tsai H. H. Deneen B. Richardson W. D. (2012). Astrocytes and disease: a neurodevelopmental perspective. Genes Dev. 26, 891–907 10.1101/gad.188326.112 22549954 PMC3347787

[B88] Montana V. Ni Y. Sunjara V. Hua X. Parpura V. (2004). Vesicular glutamate transporter-dependent glutamate release from astrocytes. J. Neurosci. 24, 2633–2642 10.1523/JNEUROSCI.3770-03.2004 15028755 PMC6729507

[B89] Morel N. Dedieu J. C. Philippe J. M. (2003). Specific sorting of the a1 isoform of the V-H^+^ATPase a subunit to nerve terminals where it associates with both synaptic vesicles and the presynaptic plasma membrane. J. Cell Sci. 116, 4751–4762 10.1242/jcs.00791 14600261

[B90] Morrow E. M. Yoo S. Y. Flavell S. W. Kim T. K. Lin Y. Hill R. S. (2008). Identifying autism loci and genes by tracing recent shared ancestry. Science 321, 218–223 10.1126/science.1157657 18621663 PMC2586171

[B91] Muro S. Garnacho C. Champion J. A. Leferovich J. Gajewski C. Schuchman E. H. (2008). Control of endothelial targeting and intracellular delivery of therapeutic enzymes by modulating the size and shape of ICAM-1-targeted carriers. Mol. Ther. 16, 1450–1458 10.1038/mt.2008.127 18560419 PMC2810502

[B92] Murthy V. N. De Camilli P. (2003). Cell biology of the presynaptic terminal. Annu. Rev. Neurosci. 26, 701–728 10.1146/annurev.neuro.26.041002.131445 14527272

[B93] Musgrove E. Seaman M. Hedley D. (1987). Relationship between cytoplasmic pH and proliferation during exponential growth and cellular quiescence. Exp. Cell Res. 172, 65–75 10.1016/0014-4827(87)90093-0 3653258

[B94] Nakamura N. Tanaka S. Teko Y. Mitsui K. Kanazawa H. (2005). Four Na^+^/H^+^ exchanger isoforms are distributed to Golgi and post-Golgi compartments and are involved in organelle pH regulation. J. Biol. Chem. 280, 1561–1572 10.1074/jbc.M410041200 15522866

[B95] Nishiguchi M. Tokugawa K. Yamamoto K. Akama T. Nozawa Y. Chaki S. (2003). Increase in secretion of glial cell line-derived neurotrophic factor from glial cell lines by inhibitors of vacuolar ATPase. Neurochem. Int. 42, 493–498 10.1016/S0197-0186(02)00139-0 12547648

[B96] Ohgaki R. Fukura N. Matsushita M. Mitsui K. Kanazawa H. (2008). Cell surface levels of organellar Na^+^/H^+^ exchanger isoform 6 are regulated by interaction with RACK1. J. Biol. Chem. 283, 4417–4429 10.1074/jbc.M705146200 18057008

[B97] Ohgaki R. Matsushita M. Kanazawa H. Ogihara S. Hoekstra D. van Ijzendoorn S. C. (2010). The Na^+^/H^+^ exchanger NHE6 in the endosomal recycling system is involved in the development of apical bile canalicular surface domains in HepG2 cells. Mol. Biol. Cell 21, 1293–1304 10.1091/mbc.E09-09-0767 20130086 PMC2847532

[B98] Ohgaki R. Van I. S. C. Matsushita M. Hoekstra D. Kanazawa H. (2011). Organellar Na^+^/H^+^ exchangers: novel players in organelle pH regulation and their emerging functions. Biochemistry 50, 443–450 10.1021/bi101082e 21171650

[B99] Ouyang Q. Lizarraga S. B. Schmidt M. Yang U. Gong J. Ellisor D. (2013). Christianson syndrome protein NHE6 modulates TrkB endosomal signaling required for neuronal circuit development. Neuron 80, 97–112 10.1016/j.neuron.2013.07.043 24035762 PMC3830955

[B100] Paradiso A. Cardone R. A. Bellizzi A. Bagorda A. Guerra L. Tommasino M. (2004). The Na^+^-H^+^ exchanger-1 induces cytoskeletal changes involving reciprocal RhoA and Rac1 signaling, resulting in motility and invasion in MDA-MB-435 cells. Breast Cancer Res. 6, R616–R628 10.1186/bcr922 15535843 PMC1064074

[B101] Pardo J. M. Cubero B. Leidi E. O. Quintero F. J. (2006). Alkali cation exchangers: roles in cellular homeostasis and stress tolerance. J. Exp. Bot. 57, 1181–1199 10.1093/jxb/erj114 16513813

[B102] Park M. Penick E. C. Edwards J. G. Kauer J. A. Ehlers M. D. (2004). Recycling endosomes supply AMPA receptors for LTP. Science 305, 1972–1975 10.1126/science.1102026 15448273

[B103] Parpura V. Zorec R. (2010). Gliotransmission: exocytotic release from astrocytes. Brain Res. Rev. 63, 83–92 10.1016/j.brainresrev.2009.11.008 19948188 PMC2862866

[B104] Perez-Palma E. Bustos B. I. Villaman C. F. Alarcon M. A. Avila M. E. Ugarte G. D. (2014). Overrepresentation of glutamate signaling in Alzheimer's disease: network-based pathway enrichment using meta-analysis of genome-wide association studies. PLoS ONE 9:e95413 10.1371/journal.pone.0095413 24755620 PMC3995778

[B105] Pescosolido M. F. Yang U. Sabbagh M. Morrow E. M. (2012). Lighting a path: genetic studies pinpoint neurodevelopmental mechanisms in autism and related disorders. Dialogues Clin. Neurosci. 14, 239–252 Available online at: http://www.dialogues-cns.org/publication/lighting-a-path-genetic-studies-pinpoint-neurodevelopmental-mechanisms-in-autism-and-related-disorders/ 23226950 PMC3513679

[B106] Peters S. U. Hundley R. J. Wilson A. K. Warren Z. Vehorn A. Carvalho C. M. (2013). The behavioral phenotype in MECP2 duplication syndrome: a comparison with idiopathic autism. Autism Res. 6, 42–50 10.1002/aur.1262 23169761 PMC3578988

[B107] Piton A. Gauthier J. Hamdan F. F. Lafreniere R. G. Yang Y. Henrion E. (2011). Systematic resequencing of X-chromosome synaptic genes in autism spectrum disorder and schizophrenia. Mol. Psychiatry 16, 867–880 10.1038/mp.2010.54 20479760 PMC3289139

[B107a] Piton A. Redin C. Mandel J. L. (2013). XLID-causing mutations and associated genes challenged in light of data from large-scale human exome sequencing. Am. J. Hum. Genet. 93, 368–383 10.1016/j.ajhg.2013.06.013 23871722 PMC3738825

[B108] Pulakat L. Cooper S. Knowle D. Mandavia C. Bruhl S. Hetrick M. (2005). Ligand-dependent complex formation between the Angiotensin II receptor subtype AT2 and Na^+^/H^+^ exchanger NHE6 in mammalian cells. Peptides 26, 863–873 10.1016/j.peptides.2004.12.015 15808917

[B109] Riess A. Rossier E. Kruger R. Dufke A. Beck-Woedl S. Horber V. (2013). Novel SLC9A6 mutations in two families with Christianson syndrome. Clin. Genet. 83, 596–597 10.1111/j.1399-0004.2012.01948.x 22931061

[B110] Rink J. Ghigo E. Kalaidzidis Y. Zerial M. (2005). Rab conversion as a mechanism of progression from early to late endosomes. Cell 122, 735–749 10.1016/j.cell.2005.06.043 16143105

[B111] Roxrud I. Raiborg C. Gilfillan G. D. Stromme P. Stenmark H. (2009). Dual degradation mechanisms ensure disposal of NHE6 mutant protein associated with neurological disease. Exp. Cell Res. 315, 3014–3027 10.1016/j.yexcr.2009.07.012 19619532

[B112] Santangelo S. L. Tsatsanis K. (2005). What is known about autism: genes, brain, and behavior. Am. J. Pharmacogenomics 5, 71–92 10.2165/00129785-200505020-00001 15813671

[B113] Santoni F. A. Makrythanasis P. Nikolaev S. Guipponi M. Robyr D. Bottani A. (2014). Simultaneous identification and prioritization of variants in familial, *de novo*, and somatic genetic disorders with VariantMaster. Genome Res. 24, 349–355 10.1101/gr.163832.113 24389049 PMC3912425

[B114] Schroer R. J. Holden K. R. Tarpey P. S. Matheus M. G. Griesemer D. A. Friez M. J. (2010). Natural history of Christianson syndrome. Am. J. Med. Genet. A 152A, 2775–2783 10.1002/ajmg.a.33093 20949524 PMC3698558

[B114a] Schuurs-Hoeijmakers J. H. Vulto-van Silfhout A. T. Vissers L. E. van de Vondervoort I. I. van Bon B. W. de Ligt J. (2013). Identification of pathogenic gene variants in small families with intellectually disabled siblings by exome sequencing. J. Med. Genet. 50, 802–811 10.1136/jmedgenet-2013-101644 24123876

[B115] Schwede M. Garbett K. Mirnics K. Geschwind D. H. Morrow E. M. (2014). Genes for endosomal NHE6 and NHE9 are misregulated in autism brains. Mol. Psychiatry. 19, 277–279 10.1038/mp.2013.28 23508127 PMC3932404

[B116] Seltzer L. E. Paciorkowski A. R. (2014). Genetic disorders associated with postnatal microcephaly. Am. J. Med. Genet. C Semin. Med. Genet. . [Epub ahead of print]. 10.1002/ajmg.c.31400 24839169

[B117] Seltzer M. M. Shattuck P. Abbeduto L. Greenberg J. S. (2004). Trajectory of development in adolescents and adults with autism. Ment. Retard. Dev. Disabil. Res. Rev. 10, 234–247 10.1002/mrdd.20038 15666341

[B118] Smith S. J. (1994). Neural signalling. Neuromodulatory astrocytes. Curr. Biol. 4, 807–810 10.1016/S0960-9822(00)00178-0 7820550

[B119] Somjen G. G. (1984). Acidification of interstitial fluid in hippocampal formation caused by seizures and by spreading depression. Brain Res. 311, 186–188 10.1016/0006-8993(84)91416-1 6488041

[B120] Stromme P. Dobrenis K. Sillitoe R. V. Gulinello M. Ali N. F. Davidson C. (2011). X-linked Angelman-like syndrome caused by Slc9a6 knockout in mice exhibits evidence of endosomal-lysosomal dysfunction. Brain 134, 3369–3383 10.1093/brain/awr250 21964919 PMC3212719

[B121] Sudhof T. C. (2008). Neuroligins and neurexins link synaptic function to cognitive disease. Nature 455, 903–911 10.1038/nature07456 18923512 PMC2673233

[B122] Sudhof T. C. (2012). The presynaptic active zone. Neuron 75, 11–25 10.1016/j.neuron.2012.06.012 22794257 PMC3743085

[B123] Sunkin S. M. Ng L. Lau C. Dolbeare T. Gilbert T. L. Thompson C. L. (2013). Allen brain atlas: an integrated spatio-temporal portal for exploring the central nervous system. Nucleic Acids Res. 41, D996–D1008 10.1093/nar/gks1042 23193282 PMC3531093

[B123a] Takahashi Y. Hosoki K. Matsushita M. Funatsuka M. Saito K. Kanazawa H. Goto Y. Saitoh S. (2011). A loss-of-function mutation in the SLC9A6 gene causes X-linked mental retardation resembling Angelman syndrome. Am. J. Med. Genet. B. Neuropsychiatr. Genet. 156B, 799–807 10.1002/ajmg.b.31221 21812100

[B124] Takeshita E. Nakagawa E. Nakatani K. Sasaki M. Goto Y. (2012). Novel AGTR2 missense mutation in a Japanese boy with severe mental retardation, pervasive developmental disorder, and epilepsy. Brain Dev. 34, 776–779 10.1016/j.braindev.2011.12.010 22269148

[B125] Tan W. H. Bird L. M. Thibert R. L. Williams C. A. (2014). If not Angelman, what is it? A review of Angelman-like syndromes. Am. J. Med. Genet. A 164A, 975–992 10.1002/ajmg.a.36416 24779060

[B126] Tarpey P. S. Smith R. Pleasance E. Whibley A. Edkins S. Hardy C. (2009). A systematic, large-scale resequencing screen of X-chromosome coding exons in mental retardation. Nat. Genet. 41, 535–543 10.1038/ng.367 19377476 PMC2872007

[B127] Tzschach A. Ullmann R. Ahmed A. Martin T. Weber G. Decker-Schwering O. (2011). Christianson syndrome in a patient with an interstitial Xq26.3 deletion. Am. J. Med. Genet. A 155A, 2771–2774 10.1002/ajmg.a.34230 21932316

[B128] Valapala M. Hose S. Gongora C. Dong L. Wawrousek E. F. Samuel Zigler J. (2013). Impaired endolysosomal function disrupts notch signalling in optic nerve astrocytes. Nat. Commun. 4, 1629 10.1038/ncomms2624 23535650 PMC3718029

[B129] van Loo K. M. Martens G. J. (2007). Genetic and environmental factors in complex neurodevelopmental disorders. Curr. Genomics 8, 429–444 10.2174/138920207783591717 19412416 PMC2647153

[B130] Vardarajan B. N. Eran A. Jung J. Y. Kunkel L. M. Wall D. P. (2013). Haplotype structure enables prioritization of common markers and candidate genes in autism spectrum disorder. Transl. Psychiatry 3, e262 10.1038/tp.2013.38 23715297 PMC3669925

[B131] Vervoort V. S. Beachem M. A. Edwards P. S. Ladd S. Miller K. E. de Mollerat X. (2002). AGTR2 mutations in X-linked mental retardation. Science 296, 2401–2403 10.1126/science.1072191 12089445

[B132] Vink J. M. Smit A. B. de Geus E. J. Sullivan P. Willemsen G. Hottenga J. J. (2009). Genome-wide association study of smoking initiation and current smoking. Am. J. Hum. Genet. 84, 367–379 10.1016/j.ajhg.2009.02.001 19268276 PMC2667987

[B133] Wagle M. Holder J. (2014). Exonic deletion of SLC9A9 in autism with epilepsy. Neurology 82:P4.338 Available online at: http://www.neurology.org/content/82/10_Supplement/P4.338 10.1212/NXG.0000000000000062 PMC4830193 27123481

[B134] Wang J. C. Foroud T. Hinrichs A. L. Le N. X. Bertelsen S. Budde J. P. (2013). A genome-wide association study of alcohol-dependence symptom counts in extended pedigrees identifies C15orf53. Mol. Psychiatry 18, 1218–1224 10.1038/mp.2012.143 23089632 PMC3752321

[B134a] Whibley A. C. Plagnol V. Tarpey P. S. Abidi F. Fullston T. Choma M. K. (2010). Fine-scale survey of X chromosome copy number variants and indels underlying intellectual disability. Am. J. Hum. Genet. 87, 173–188 10.1016/j.ajhg.2010.06.017 20655035 PMC2917707

[B135] Williams C. A. Lossie A. Driscoll D. (2001). Angelman syndrome: mimicking conditions and phenotypes. Am. J. Med. Genet. 101, 59–64 10.1002/ajmg.1316 11343340

[B136] Xinhan L. Matsushita M. Numaza M. Taguchi A. Mitsui K. Kanazawa H. (2011). Na^+^/H^+^ exchanger isoform 6 (NHE6/SLC9A6) is involved in clathrin-dependent endocytosis of transferrin. Am. J. Physiol. Cell Physiol. 301, C1431–C1444 10.1152/ajpcell.00154.2011 21881004

[B137] Xu L. M. Li J. R. Huang Y. Zhao M. Tang X. Wei L. (2012). AutismKB: an evidence-based knowledgebase of autism genetics. Nucleic Acids Res. 40, D1016–D1022 10.1093/nar/gkr1145 22139918 PMC3245106

[B138] Zanni G. Barresi S. Cohen R. Specchio N. Basel-Vanagaite L. Valente E. M. (2014). A novel mutation in the endosomal Na^+^/H^+^ exchanger NHE6 (SLC9A6) causes Christianson syndrome with electrical status epilepticus during slow-wave sleep (ESES). Epilepsy Res. 108, 811–815 10.1016/j.eplepsyres.2014.02.009 24630051

[B139] Zerial M. McBride H. (2001). Rab proteins as membrane organizers. Nat. Rev. Mol. Cell Biol. 2, 107–117 10.1038/35052055 11252952

[B140] Zhang-James Y. Dasbanerjee T. Sagvolden T. Middleton F. A. Faraone S. V. (2011). SLC9A9 mutations, gene expression, and protein-protein interactions in rat models of attention-deficit/hyperactivity disorder. Am. J. Med. Genet. B Neuropsychiatr. Genet. 156B, 835–843 10.1002/ajmg.b.31229 21858920 PMC3168688

[B141] Zhang-James Y. Middleton F. A. Sagvolden T. Faraone S. V. (2012). Differential expression of SLC9A9 and interacting molecules in the hippocampus of rat models for attention deficit/hyperactivity disorder. Dev. Neurosci. 34, 218–227 10.1159/000338813 22777493 PMC3480220

